# Associations and mediators between vitiligo and cardiovascular diseases: a Mendelian randomization study

**DOI:** 10.1038/s41598-025-95638-y

**Published:** 2025-04-01

**Authors:** Xiaoyan Zhang, Lei Pu, Cheng Pu, Qian He

**Affiliations:** 1 The Key Laboratory of Adolescent Health Assessment and Exercise Intervention of the Ministry of Education, East China Normal University, Shanghai, 200241 People’s Republic of China; 2 School of Martial Arts, Shanghai University of Sport, Shanghai, 200438 People’s Republic of China; 3 Preventive Medicine Department, Suzhou Wujiang District Second People’s Hospital, 999 DaChun Road, Suzhou, 215221 Jiangsu People’s Republic of China

**Keywords:** Mendelian randomization, Vitiligo, Cardiovascular diseases, Inflammatory cytokines, Causal association, Genetics, Cardiology

## Abstract

Previous studies yielded conflicting results on the associations between vitiligo and cardiovascular diseases (CVD), and its underlying mechanisms remain unclear. This MR study aims to elucidate the underlying causal association between vitiligo and the risk of CVD, as well as the potential mediator. Using summary statistics from genome-wide association study (GWAS), we conducted a bidirectional two-sample Mendelian randomization (MR) analysis and mediation analysis to investigate the causal association between vitiligo and 13 CVD outcomes and potential mediators. The IVW method was the main MR analysis method, supplemented by MR-Egger, weighted median, and weighted mode methods. Multiple sensitivity analyses were applied to enhance the robustness of the results. Bioinformatics analysis involved Protein-Protein Interaction (PPI) network, Gene Ontology (GO) and GeneMANIA functional analysis analysis. MR analysis indicated that genetically predicted vitiligo was significantly associated with higher risk of coronary heart disease (CHD) (OR = 1.0199, 95% CI = 1.0024–1.0377, *p* = 0.026). There was no significant causal association between vitiligo and 12 other cardiovascular diseases. And reverse MR analysis found no causal effect of CVD on vitiligo. CCL11 was identified to partially mediate the association between vitiligo and CHD. GO and GeneMANIA suggested that CCL11 may mediate the association between vitiligo and CHD through chemokine-related functions and pathways. Our study revealed the potential causal association between vitiligo and CHD, with CCL11 as a potential mediator. Further studies are necessitated to elucidate the exact association and the mechanisms.

**Supplementary Information:**

The online version contains supplementary material available at 10.1038/s41598-025-95638-y.

## Introduction

Classified as a common autoimmune disease, vitiligo is a depigmenting disorder characterized by white patches on the skin due to selective loss of melanocytes, with a global prevalence rate of 0.5–2%. Accumulating epidemiological evidence has recognized vitiligo as a systemic disease closely associated with cardiovascular diseases (CVD)^1^, in which inflammation may play a role. However, the causal associations between the three remain largely unclear.

Previous studies provided inconsistent results regarding the association between vitiligo and CVD. Some case-control studies revealed increased risk of CVD in patients with vitiligo^2–4^. And the presence of vitiligo was found to be significantly correlated with a family history of CVD^5^. However, Rodriguez-Marti et al. suggested that vitiligo patients exhibited fewer CVD risk factors^6^. A cohort study by Wu et al. found no significant association between vitiligo and major adverse cardiovascular events^7^. As such, further verification of the association between vitiligo and CVD is necessitated. However, given that current studies are mostly observational and limited in sample size, they are susceptible to reverse causality and potential confounding factors such as population specificity and data collection methods. And it remains difficult to establish definite causal relationship between vitiligo and CVD.

Moreover, the pathophysiological mechanisms underlying the association between vitiligo and CVD remain unclear. Studies have suggested that vitiligo and CVD share certain pathophysiological pathways. For instance, inflammatory response is identified as a contributor to vitiligo as well as a risk factor for CVD. And studies have suggested that some proinflammatory cytokines that are generally higher in vitiligo patients, such as tumor necrosis factor (TNF)-α, interleukin (IL)-1 and IL-6, are also involved in the development of atherosclerosis^8,9^. Given the role of inflammation in both vitiligo and CVD, we postulated that inflammatory cytokines may have potential mediating role between vitiligo and CVD.

Mendelian randomization (MR) is an epidemiological research method to infer the causal relationship between exposure and outcome. It uses the genetic variant as instrumental variable (IV) to infer the causal effect of exposure on the outcome. According to Mendel’s second law of heredity, determined at conception, genetic variation precedes the development of diseases and is independent of environmental factors. Therefore, compared to cross-sectional observational studies, MR is less prone to confounding factors and can minimize the possibility of reverse causality.

In this study, we conducted a bidirectional two-sample MR study to determine the causal relationship between vitiligo and the risk of CVD. Subsequently, we performed a mediation analysis to assess the potential mediating effects of inflammatory cytokines. Ultimately, we performed bioinformatics analysis to probe into the role of the identified inflammatory cytokine in cellular pathways and functions.

## Results

### Causal association of vitiligo with CVD

After strict quality control, we selected a total of 42 SNPs significantly and independently associated with vitiligo as IVs. And the F-statistic values were all above 10 (Supplementary Table S3).

The results indicated that genetically predicted vitiligo was causally associated with only 1 CVD outcome, namely coronary heart disease (CHD). Specifically, vitiligo was associated with increased risk of CHD (OR = 1.021, 95% CI = 1.003–1.0389, *p* = 0.015). IVW, MR-Egger, weighted median, and weighted mode methods all showed consistent directions (Supplementary Table S4; Supplementary Fig. S1).

However, no significant causal relationship was found between genetically predicted vitiligo and the other 12 CVD outcomes, including aortic aneurysm (OR = 0.9871, *p* = 0.429; Supplementary Fig. S2), atrial fibrillation (OR = 0.9913, *p* = 0.207; Supplementary Fig. S3), endocarditis (OR = 0.9588, *p* = 0.199; Supplementary Fig. S4), heart failure (OR = 1.00006, *p* = 0.584; Supplementary Fig. S5), hypertrophic cardiomyopathy (OR = 1.0033, *p* = 0.925; Supplementary Fig. S6), hypertension (OR = 1.0001, *p* = 0.114; Supplementary Fig. S7), myocardial infarction (OR = 1.0095, *p* = 0.352; Supplementary Fig. S8), myocarditis (OR = 1.0224, *p* = 0.426; Supplementary Fig. S9), non-rheumatic valvular disease (OR = 0.9983, *p* = 0.839; Supplementary Fig. S10), pericarditis (OR = 1.0583, *p* = 0.141; Supplementary Fig. S11), peripheral arterial disease (OR = 0.9999, *p* = 0.245; Supplementary Fig. S12), and stroke (OR = 1.0055, *p* = 0.556; Supplementary Fig. S13). MR-Egger, weighted median, and weighted mode methods all showed consistent directions with IVW.

### Reverse MR analysis of vitiligo and CVD

In reverse MR analysis, each CVD outcome was taken as the exposure and vitiligo as the outcome. We extracted SNPs for each 13 CVD outcome respectively (*p* < 5 × 10^−8^ or *p* < 5 × 10^−6^, F>10). And MR analysis showed no significant causal effect of CVD on vitiligo (Supplementary Table S5). After removing the detected outliers, there was still no reverse causality (Supplementary Table S6).

### Sensitivity analysis

**Fig. 1:**
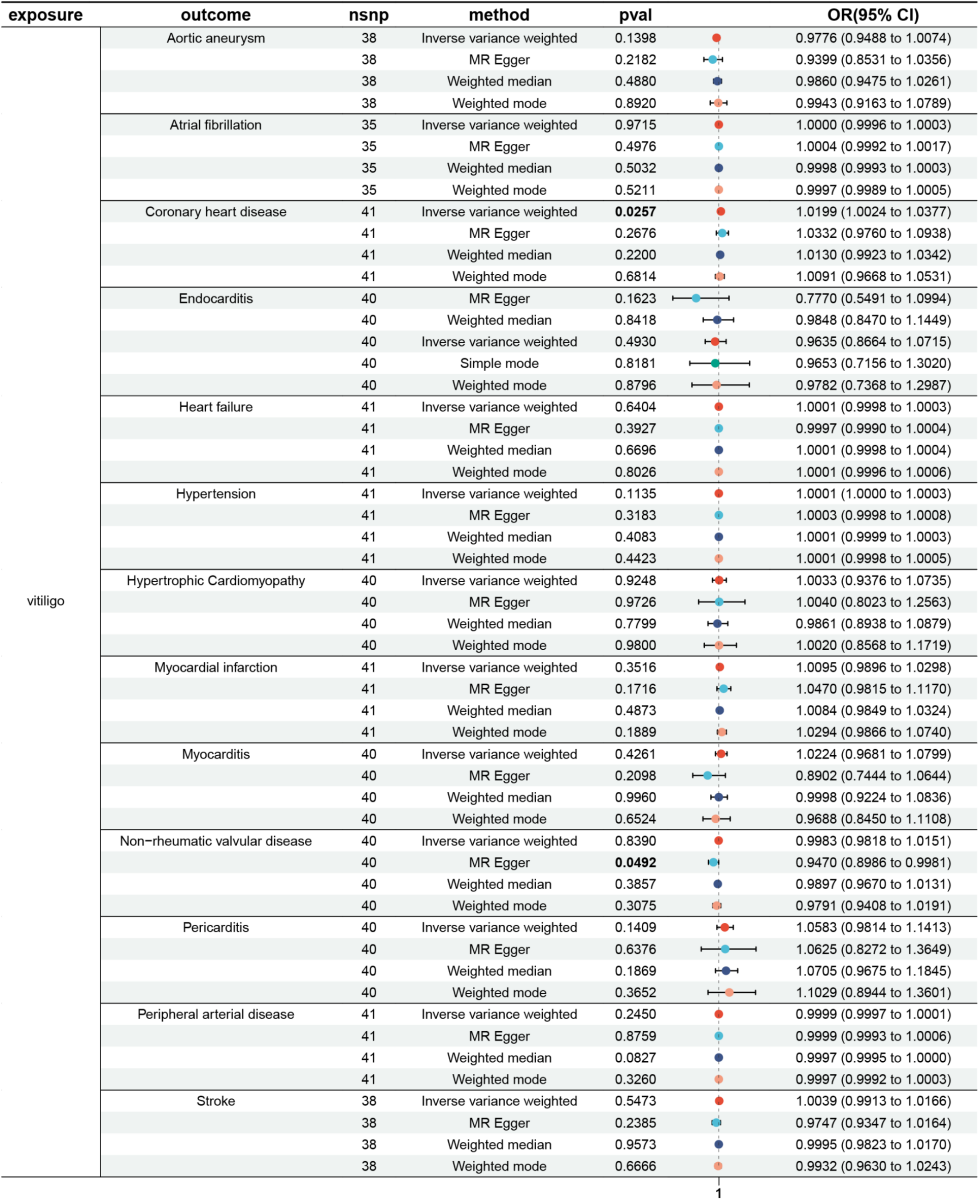
Associations between vitiligo and 13 CVD outcomes.

Multiple sensitivity analyses were conducted to assess heterogeneity and pleiotropy in causal effects of vitiligo and CVD (Supplementary Table S7). Cochran’s Q test showed that CHD (*p* = 0.036), myocardial infarction (*p* = 0.012), stroke (*p* < 0.001), aortic aneurysm (*p* = 0.008) had heterogeneity, but no significant pleiotropy was found. MR-Egger intercept test indicated no horizontal pleiotropy. The MR-PRESSO method detected the outliers (Vitiligo and CHD: rs11720951; vitiligo and myocardial infarction: rs4766897; vitiligo and stroke: rs159960, rs4766897; vitiligo and aortic aneurysm: rs4766897, rs6573910), but there was no change in the results after the outliers were removed (Supplementary Table S8). Specifically, vitiligo was still significantly associated with CHD (OR = 1.02, 95% CI = 1.002–1.038, *p* = 0.026), while other CVD were not (Fig. 1).

However, after adjusting for multiple testing using Bonferroni correction, we found that the association between vitiligo and CHD was no longer significant (*p* = 0.3; Supplementary Table S8). Therefore, we performed a validation analysis in another external replication cohort to evaluate the robustness of the results. The replication results showed that vitiligo was still significantly associated with CHD (OR = 1.02, 95% CI = 1.003–1.031, *p* = 0.014; Supplementary Table S9). The consistent results in both cohorts suggested a potential causal association between vitiligo and CHD.

### Causal association of mediators with CHD

To further explore the mechanism underlying the potential causal association between vitiligo and CHD, we performed a two-step mediation MR analysis with inflammatory cytokines as mediators. To ensure sufficient SNPs for mediation analysis, we applied a threshold of 1e-05, which was extensively used in previous studies. Additionally, a less stringent threshold can incorporate more available IVs, allow for measurement errors, and identify potential biological associations. Finally, we identified 2832 SNPs as IVs for 91 inflammatory cytokines (F>10).

The IVW method suggested that 11 inflammatory cytokines were causally associated with CHD, all of which were included for further mediation analysis as possible mediators (Supplementary Table S10). IVW, MR-Egger, weighted median, and weighted mode methods all showed consistent directions.

### Causal association of vitiligo with mediators

Following our analysis of the effect of mediators on CHD, we then assessed the causal effects of genetically predicted vitiligo on the abovementioned mediators.

**Fig. 2:**
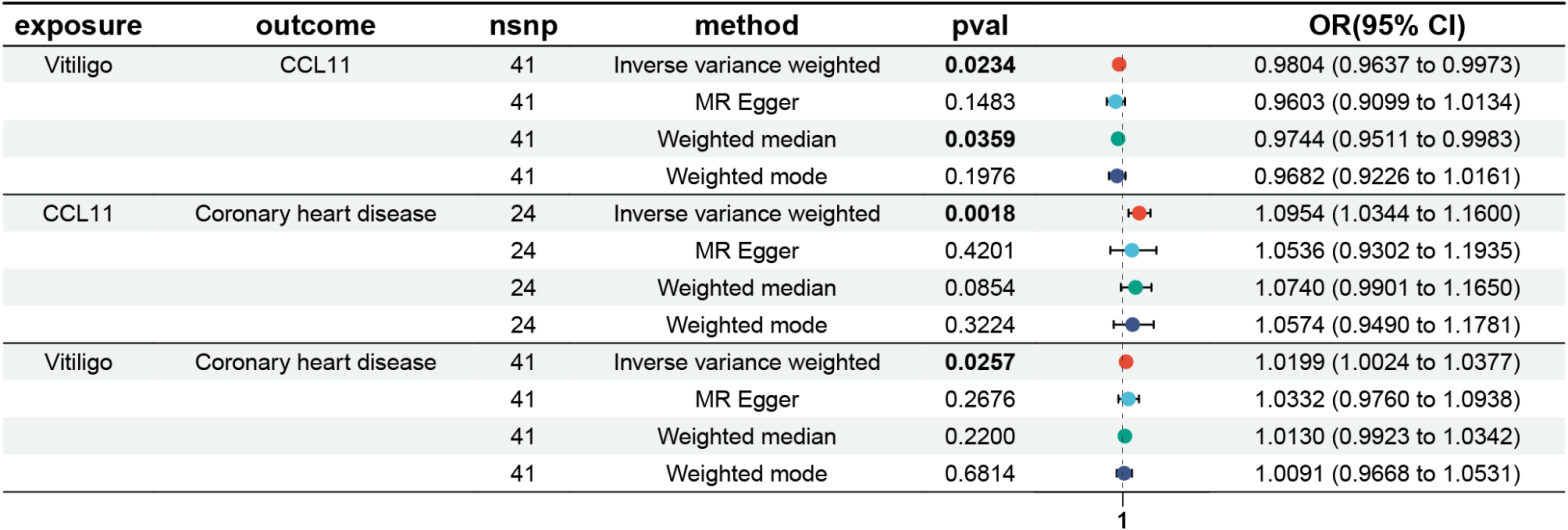
Mediation analysis.

Among the 11 inflammatory cytokines associated with CHD, vitiligo was found to be causally associated with one of them, namely CCL11 (OR = 0.9804, 95% CI 0.9637–0.9973, *p* = 0.023) (Supplementary Fig. S14). And the estimation direction of the other three methods, MR-Egger, weighted median, and weighted mode, were all consistent with that of IVW (Fig. 2).

### Mediation proportion

We identified CCL11 as the potential mediator in the causal pathway from vitiligo to CHD. Specifically, vitiligo was negatively associated with CCL11 (OR = 0.9804, 95% CI 0.9637–0.9973, *p* = 0.023), which was in turn associated with an increased risk of CHD (OR = 1.0954, 95% CI 1.0344–1.1600, *p* = 0.002) (Supplementary Fig. S15). Combined, CCL11 negatively mediated the association between vitiligo and CHD, with a mediation proportion of −14.3% (95% CI −0.00369, −0.0000114, *p* = 0.0486).

### Bioinformatics analysis

**Fig. 3:**
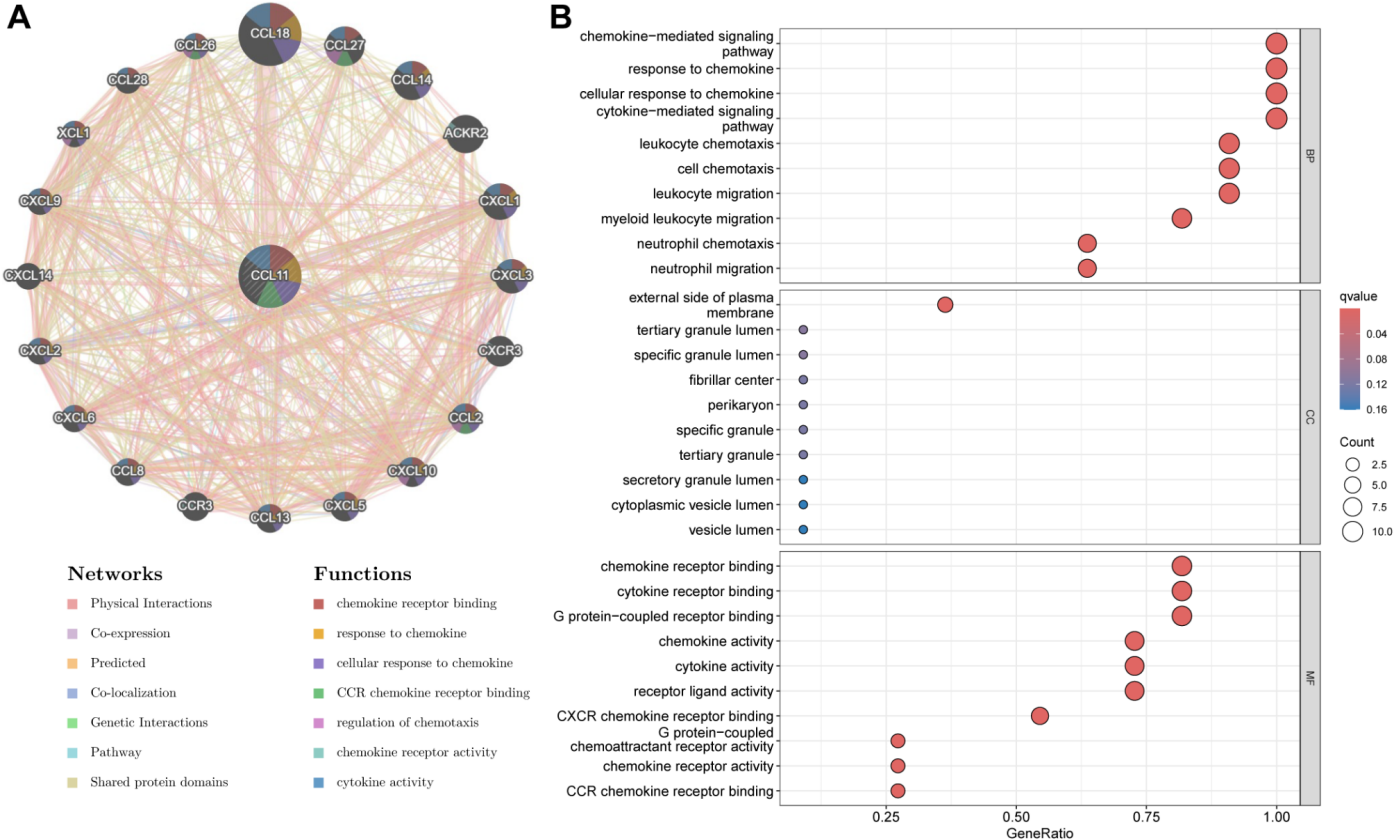
Analysis of potential functions and pathways of CCL11. (**A**) GeneMANIA functional analysis. (**B**) GO enrichment analysis.

First, we used the STRING database to construct a PPI network based on CCL11, and identified its interactions with 10 proteins (Supplementary Table S11). Subsequently, we performed GO and GeneMANIA functional analysis on CCL11 and 10 proteins (Fig. 3). GO enrichment analysis suggested that the most significant BP included chemokine-mediated signaling pathway, response to chemokine, and cellular response to chemokine (Supplementary Table S12). GeneMANIA functional analysis revealed that these genes were mainly involved in chemokine receptor binding, response to chemokine, cellular response to chemokine, etc.

## Discussion

To the best of our knowledge, this is the first MR study to report the causal association between vitiligo and CVD. In this bidirectional two-sample and two-step MR analyses, we examined the causal relationship and potential mediators between vitiligo and 13 CVD outcomes. The results revealed that there was a potential causal association between genetically predicted vitiligo and an increased risk of CHD. And there was no reverse causality found between vitiligo and CVD. Meanwhile, our two-step mediation MR analysis found that the causal association between vitiligo and CHD was partially mediated by CCL11. PPI network identified the interaction of CCL11 with 10 proteins, which were mostly CXCL chemokines and CCR receptors. GO and GeneMANIA functional analysis suggested that CCL11 was mainly involved in chemokine-related signaling pathway, response to chemokine, and cellular response to chemokine.

After applying Bonferroni correction, we found no significant association between vitiligo and 13 CVD. Bonferroni correction reduces the risk of Type I errors (false positives) by dividing the significance level by the number of tests. However, its stringent threshold may lead to a higher chance of Type II errors (false negatives), and thus fail to detect true effects that are actually present, especially when the tests are not independent^10^. Therefore, we examined the association between vitiligo and CHD in the replication cohort. The replication analysis found a significant association between vitiligo and CHD, indicating there might be a Type II error that potentially underestimated the causal association. Although replication results supported this association, it still needs to be interpreted with caution since Bonferroni correction did not reach the significance level. The identified causal association between vitiligo and CHD was potential but still meaningful.

Limited studies have explored the causal association between vitiligo and CHD, but the adverse effect of vitiligo on risk of CHD has been confirmed by previous findings from traditional observational studies. Coronary atherosclerotic heart disease, also called coronary heart disease or coronary artery disease, is characterized by atherosclerosis in the coronary arteries. Atherosclerosis have been recognized as a chronic inflammatory disease predisposing to CHD, while carotid intima media thickness (CIMT) and atherosclerotic plaques are considered as hallmarks of atherosclerosis and independent predictors of cardiovascular events^11^. A cross-sectional, case-control study by Azzazi et al. found that CIMT and the presence of atherosclerotic plaques were significantly increased in vitiligo patients^3^. This suggested that vitiligo was associated with increased risk of atherosclerotic cardiovascular diseases, which was in line with our findings. Similar observation was made in another study that determined vitiligo as an independent predictor of subclinical atherosclerosis^4^. A recent large-scale retrospective study also reported that patient with vitiligo displayed an increased risk of CVD, including CHD^12^. Mechanistically, studies have suggested that vitiligo patients had higher incidence of metabolic syndrome, which in turn increased the risk of CVD^13–15^. Additionally, vitiligo patients had higher levels of homocysteine, while hyperhomocysteinaemia has been identified as an independent risk factor for multiple cardiovascular diseases including CHD^16,17^. Our MR analysis provides genetic evidence supporting the association between vitiligo and the risk of CHD and further corroborates the causality. More exploratory studies are necessitated to validate the association and elucidate the underlying mechanisms.

In contrast to our results, a few studies have noted associations between vitiligo and some of the 12 CVD outcomes. A meta-analysis confirmed the association between vitiligo and hypertension, with a prevalence ranging from 7.0 to 34.0%^18^. A case-control study suggested that vitiligo patients, particularly those with autoimmune diseases, were more disposed to myocardial infarction^2^. Several case reports presented cases of vitiligo associated with pernicious anemia^19,20^. However, our MR analysis demonstrated no causal associations. The observed associations in observational studies could be affected by limited sample size and potential confounding such as ethnicity and comorbidities.

Eotaxin-1 (CCL11) is a member of CC chemokine family and mainly facilitates the selective recruitment of eosinophils to inflammatory sites by binding to CCR3. Our study revealed the negative mediating role of CCL11 in the causal association between vitiligo and CHD. There is limited direct evidence regarding the negative correlation between CCL11 and vitiligo. Nonetheless, studies have shown that Th1-derived cytokine IFN-γ is induced in human vitiligo and highly expressed in the lesional skin^21–26^. Meanwhile, IFN-γ has been found to suppress the recruitment of eosinophils and inhibit the production of eotaxin^27–30^. Similarly, CCL11 binds with high affinity to CXCR3, while the expression of CXCR3 is increased in vitiligo^31,32^. It is likely that CXCR3 act as a decoy receptor that sequesters locally produced CCL11 and downregulates its responses at CCR3-expressing cells^32^. Taken together, these studies indicate that vitiligo may be indirectly associated with decreased CCL11 levels, as our findings suggested.

Meanwhile, the mediation analysis also revealed that CCL11 was associated with a higher risk of CHD, which is consistent with previous research on the pathophysiological role of CCL11 in coronary atherosclerotic heart disease. CCL11 has been proven to be overexpressed in the atherosclerotic plaques and participate in vascular inflammation through CCR3 on vascular smooth muscle cells (SMCs)^33^. Circulating CCL11 levels are also increased in patients with coronary artery disease^34,35^. Clinical studies have noted a significant association of CCL11 levels with the presence and extent of coronary artery disease^36^. Additionally, CCL11 has also been shown to induce the migration of SMCs from the arterial media to the intima, and induce angiogenic responses in atherogenesis, which would contribute to the onset and progression of atherosclerosis^37–39^. According to our bioinformatics analysis, the interaction network of CCL11 was mostly involved in the chemokine-related biological function and signaling pathway, while chemokines are extensively involved in all stages of atherosclerosis from accumulation of fatty streaks to formation of atherosclerotic plaque^40,41^. Therefore, the downregulation of CCL11 in vitiligo had a partial protective effect on the development of CHD. The inhibition or downregulation of CCL11 might be a potential therapeutic target for CHD in patients with vitiligo.

As opposed to the positive total effect of vitiligo on the risk of CHD, CCL11 negatively mediated this association and attenuated the effect of vitiligo on CHD. The effect of CCL11 herein could also be interpreted as suppression effect. Nevertheless, the total effect of vitiligo on the risk of CHD includes any effect through potential mediators, which involve multiple biological mechanisms accordingly. There could be other larger or more significant mediating effects or pathways associated with a higher risk of CHD that require further investigation.

Interestingly, phototherapy, an established treatment for cutaneous diseases, have shown cardiovascular benefits in treating vitiligo. Some experimental studies suggested that narrowband UVB phototherapy could decrease the risk of cardiovascular events in vitiligo patients, and prevent and stabilize atherosclerosis via the suppression of inflammatory Th1/Th17 responses^42–44^. It is necessary for more research to investigate the effects of other vitiligo treatments on the prevention of CVD.

Our study boasted several strengths. Firstly, we first adopted MR analysis to explore the potential causal associations between vitiligo and several CVD outcomes, which can largely avoid the main limitations of observational design, such as confounding factors and reverse causality. Secondly, the use of summary-level data from GWAS greatly increased the statistical power in assessing causal effects. Thirdly, the consistency of multiple methods, namely the IVW, weighted median, MR-Egger, and weighted mode, as well as a series of sensitivity analyses enhanced the reliability and robustness of our findings. Additionally, we not only investigated the causal association, but also delved into the analysis of the potential mediator as well as the functions and pathways, which could enrich the current understanding of the association between vitiligo and CHD.

However, our study had its limitations. Firstly, the study population was predominantly of European ancestry, which might give rise to ethnic biases and restrict the generalizability and applicability of our conclusions to other ethnic groups. Future studies are necessitated to include more diverse populations. Secondly, despite multiple sensitivity analyses conducted, there was no guarantee that biases could be completely eliminated. Thirdly, for lack of individual-level data, we failed to consider the possibility of stratification effects and explore the causal association in subgroups based on the type, duration or activity of vitiligo. Additionally, the causal effect of vitiligo on CVD may be mediated or moderated by other mediators, which necessitates more research to quantify other potential mediators and confirm our findings. Finally, the relationship between vitiligo and CVD is far more complex than a linear relationship, requiring consideration of potential nonlinear effects and interactions with other factors. For example, the MR framework assumes that genetic instruments are independent of environmental confounders; however, although we found no significant horizontal pleiotropy in IVs, it is difficult to completely avoid the interactions with environmental factors in MR design, such as dietary patterns or UV exposure. Future studies may benefit from multivariable, MR gene-environment interaction analysis, or more complex statistical models to account for more confounding factors.

In the future, advanced computer vision algorithms could be leveraged to automate the analysis of imaging data, providing deeper insights into the relationship between vitiligo and CVD. For instance, techniques like DeepLab and Feature Pyramid Networks (FPN) are designed for image segmentation and object detection. DeepLab is able to capture spatial features at multiple scales, and segment objects with high accuracy even in complex scenes; FPN utilized a feature pyramid and a top-down architecture to detect objects at multiple scales with improved accuracy^45,46^. These advanced computer vision architectures are particularly useful in medical imaging, such as detecting lesions or plaques, and extracting metrics like carotid intima-media thickness. The integration of multimodal data with genetic insights may help uncover novel patterns and strengthen mechanistic understanding of how vitiligo contributes to the increased risk of CHD.

## Conclusions

In summary, our study identified the potential causal association between vitiligo and CHD, and highlighted the mediating effect of CCL11 in this association. Further studies with laboratory data and longitudinal observations are necessitated to elucidate the exact association and mechanisms. These insights extend the existing body of knowledge and inform the clinical management of the two diseases.

## Method

### Study design

**Fig. 4:**
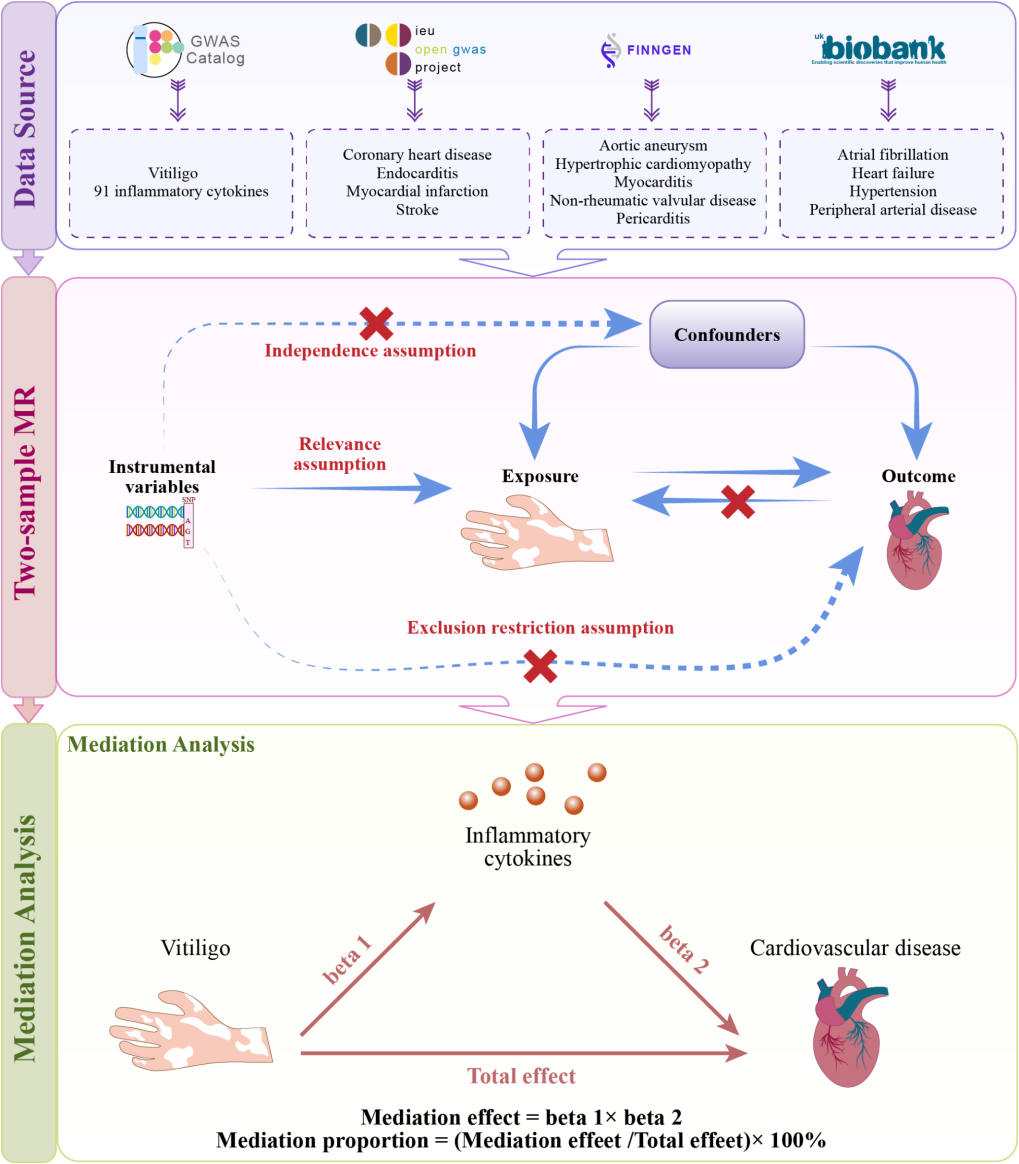
The research flowchart.

Fig. 4 presents the research flowchart. In this study, a bidirectional two-sample MR analysis was first conducted to infer the causal relationship between vitiligo and CVD, with vitiligo as the exposure and CVD as the outcome. Subsequently, a two-step mediation MR analysis was performed with 91 inflammatory cytokines as mediators. Ultimately, GO and GeneMANIA functional analyses were performed on the identified inflammatory cytokine to reveal the potential functions of the mediators. This MR study strictly followed STROBE-MR guidelines and were self-reviewed (Supplementary Table S1).

MR analysis is based on the following three core assumptions: (1) the relevance assumption: the included IVs are strongly associated with the exposure; (2) the independence assumption: IVs are independent of any confounders of the association between the exposure and outcome; (3) the exclusion restriction assumption: IVs do not directly affect the outcome, but only through the exposure.

### Data sources

All the summary statistics were obtained from publicly available meta-analyses of GWAS, which all obtained informed consent and ethical approval in the original study. To minimize biased estimates caused by ethnic stratification, the population was limited to European ancestry (Supplementary Table S2).

Summary statistics for vitiligo were derived from a recent GWAS meta-analysis by Jin et al. (http://ftp.ebi.ac.uk/pub/databases/gwas/summary_statistics/GCST004001-GCST005000/GCST004785/), which had the largest case size in the vitiligo GWAS study thus far (4,680 cases and 39,586 controls)^47^. We incorporated summary-level data for 13 CVD outcomes, including aortic aneurysm, atrial fibrillation, coronary heart disease, endocarditis, heart failure, hypertrophic cardiomyopathy, hypertension, myocardial infarction, myocarditis, non-rheumatic valvular disease, pericarditis, peripheral arterial disease, and stroke, each of which was used as the outcome in turn.

The GWAS data of 91 inflammatory cytokines were from the GWAS Catalog, (https://www.ebi.ac.uk/gwas/studies/GCsT90274758-GCST90274848), including 14,824 European ancestry individuals^48^.

### Instrumental variable selection

To satisfy the three assumptions, the following criteria were used to select IVs. First, the genome-wide significance threshold of *p* < 5 × 10^−8^ was applied to select SNPs significantly associated with exposure. If few SNPs were identified, a less stringent threshold of *p* < 5 × 10^−6^ was applied instead. Second, SNPs were clumped (r^2^ = 0.001, kb = 10,000) to avoid strong linkage disequilibrium (LD) and ensure that the SNPs were independent of each other. Third, *F* statistic was used to calculate the strength of each IV by $\:F=\frac{N-K-1}{K}\times\:\frac{{R}^{2}}{1-{R}^{2}}$ (N= the sample size, K= the number of IVs, R^2^= the proportion of variance of exposure explained by SNPs). SNPs with F<10 were deemed weak IVs and were removed to exclude weak instrument bias. Additionally, to make sure each SNP was associated with the same effect allele, exposure and outcome summary statistics were harmonized, and palindromic and ambiguous SNPs were removed.

### Two-sample MR analysis

The IVW method was the main MR analysis method, supplemented by MR-Egger, weighted median, and weighted mode methods.

The IVW can combine the Wald ratio estimates of effects from each SNP to obtain the overall weighted estimates, providing robust causal estimates in the absence of horizontal pleiotropy. However, since IVW presupposes that all SNPs are valid and independent of each other, we used several supplementary methods, and their consistent direction of estimates can enhance the validity and robustness of the IVW results. Specifically, MR-Egger regression can provide unbiased results in the presence of potential pleiotropy; the weighted median estimates can provide valid estimates when at least 50% of the weight are from valid IVs; and the weighted mode method has less strict assumptions and lower efficacy^49^.

### Reverse Mendelian randomization analysis

To explore the causal association of CVD with vitiligo, each CVD outcome was used as the exposure, the related SNPs were used as IVs, and vitiligo was used as the outcome. The analysis process of reverse causality was consistent with that of the causal association of vitiligo with CVD.

### Mediation analysis

We further conducted a two-step mediation analysis to assess whether inflammatory cytokines mediated the causal pathway from vitiligo to CVD. β1* β2 represented the mediating effect (indirect effect) of vitiligo on CVD via mediators, where β1 represented the effect of vitiligo on mediators and β2 the effect of mediators on CVD. The total effect (β0) referred to the causal effect of vitiligo on CVD. And the direct effect of vitiligo on CVD was calculated by β0-(β1*β2). Proportion mediated was calculated by (β1*β2)/β0. Standard errors and CIs were calculated by the delta method.

### Sensitivity analysis

A number of sensitivity analyses were conducted to assess the validity of the results. Specifically, the Cochran’s Q test was used to quantify the heterogeneity between IVs; the MR-Egger intercept was utilized to detect pleiotropy; MR pleiotropy residual sum and outlier (MR-PRESSO) was employed to identify and correct for pleiotropy and significant outliers; leave-one-out analyses were conducted to determine the effect of each single SNP on the overall causal estimate. The scatter plots, forest plots, and funnel plots were used for sensitivity analysis. In addition, we use a replication cohort to validate the significant results to avoid false positives caused by the datasets.

### Statistical analysis

All analyses were performed with the “TwoSampleMR” (version 0.5.6), “MR-PRESSO”, and “mr.raps” packages in R software (version 4.2.1). All causal estimates were expressed as odds ratios (OR) and 95% confidence intervals (IV). And “reshape2”, “circlize”, “ComplexHeatmap” packages were used for visualization. *p* < 0.05 was considered potentially significant; Bonferroni-corrected *p* < 0.05 was considered strongly significant.

### Functional enrichment analysis

To further reveal the role of the mediator, we used the STRING database (https://string-db.org/) to retrieve the identified protein and constructed a Protein-Protein Interaction (PPI) network. Additionally, we also investigated the functional pathways of the identified protein and the associated proteins by utilizing GeneMANIA (http://www.genemania.org). Gene Ontology (GO) enrichment analysis was employed to uncover significant biological processes (BP).

## Electronic supplementary material

Below is the link to the electronic supplementary material.

Supplementary Material 1

Supplementary Material 2

## Data Availability

The datasets used and/or analyzed during the current study available from the corresponding author on reasonable request.

## References

[1] Conrad, N. et al. Autoimmune diseases and cardiovascular risk: A population-based study on 19 autoimmune diseases and 12 cardiovascular diseases in 22 million individuals in the UK. *Lancet* **400**(10354), 733–743 (2022). 36041475 10.1016/S0140-6736(22)01349-6

[2] Ahmed, A. R. S., Hussein, M. S. & Mansour, A. I. Are patients with vitiligo more prone to myocardial infarction?: A case-control study. *J. Clin. Aesthet. Dermatol.* **12**(11), 28–31 (2019). PMC6937161 32038754

[3] Azzazi, Y. et al. Support for increased cardiovascular risk in non-segmental vitiligo among Egyptians: A hospital-based, case-control study. *Pigment Cell. Melanoma Res.* **34**(3), 598–604 (2021). 33098225 10.1111/pcmr.12941

[4] Namazi, N. et al. Increased risk of subclinical atherosclerosis and metabolic syndrome in patients with vitiligo: A real association or a coincidence? *Dermatol. Ther.* **34**(2), e14803 (2021). 33496053 10.1111/dth.14803

[5] Arunachalam, M. et al. Non-segmental vitiligo and psoriasis comorbidity—A case-control study in Italian patients. *J. Eur. Acad. Dermatol. Venereol.* **28**(4), 433–437 (2014). 23441884 10.1111/jdv.12117

[6] Rodriguez-Martin, M. et al. Patients with vitiligo present fewer cardiovascular risk factors: Results from a case-control study. *J. Eur. Acad. Dermatol. Venereol.* **27**(1), 124–125 (2013). 22176574 10.1111/j.1468-3083.2011.04392.x

[7] Wu, P. C. et al. Lack of association between vitiligo and major adverse cardiovascular events: A population-based cohort study. *J. Eur. Acad. Dermatol. Venereol.* **37**(6), e773–e775 (2023). 36734285 10.1111/jdv.18936

[8] Gholijani, N., Yazdani, M. R. & Dastgheib, L. Predominant role of innate pro-inflammatory cytokines in vitiligo disease. *Arch. Dermatol. Res.* **312**(2), 123–131 (2020). 31620869 10.1007/s00403-019-01996-9

[9] Seremet, S. & Gurel, M. S. Miscellaneous skin disease and the metabolic syndrome. *Clin. Dermatol.* **36**(1), 94–100 (2018). 29241760 10.1016/j.clindermatol.2017.09.016

[10] Perneger, T. V. What’s wrong with bonferroni adjustments. *BMJ* **316**(7139), 1236–1238 (1998). 9553006 10.1136/bmj.316.7139.1236 PMC1112991

[11] Katakami, N., Matsuoka, T. A. & Shimomura, I. Clinical utility of carotid ultrasonography: Application for the management of patients with diabetes. *J. Diabetes Investig.* **10**(4), 883–898 (2019). 30884192 10.1111/jdi.13042 PMC6626964

[12] Fraczek, A. et al. Vitiligo is associated with an increased risk of cardiovascular diseases: A large-scale, propensity-matched, US-based retrospective study. *EBioMedicine* **109**, 105423 (2024). 39461193 10.1016/j.ebiom.2024.105423 PMC11543909

[13] Atas, H. & Gonul, M. Increased risk of metabolic syndrome in patients with vitiligo. *Balkan Med. J.* **34**(3), 219–225 (2017). 28443562 10.4274/balkanmedj.2016.1005 PMC5450861

[14] Pietrzak, A. et al. Metabolic syndrome in vitiligo. *Dermatol. Ther.* **25**(Suppl 1), S41–S43 (2012). 23237037 10.1111/dth.12012

[15] Sharma, Y. K. et al. Metabolic syndrome in vitiligo patients among a semi-urban Maharashtrian population: A case control study. *Diabetes Metab. Syndr.* **11**(Suppl 1), S77–S80 (2017). 28017282 10.1016/j.dsx.2016.12.009

[16] Karadag, A. S. et al. Serum holotranscobalamine, vitamin B12, folic acid and homocysteine levels in patients with vitiligo. *Clin. Exp. Dermatol.* **37**(1), 62–64 (2012). 22182436 10.1111/j.1365-2230.2011.04142.x

[17] Silverberg, J. I. & Silverberg, N. B. Serum homocysteine as a biomarker of vitiligo vulgaris severity: A pilot study. *J. Am. Acad. Dermatol.* **64**(2), 445–447 (2011). 21238838 10.1016/j.jaad.2010.08.025

[18] Kang, P. et al. Association between vitiligo and relevant components of metabolic syndrome: A systematic review and meta-analysis. *J. Dtsch. Dermatol. Ges*. **20**(5), 629–641 (2022). 35499212 10.1111/ddg.14717

[19] Held, J. L. & Kohn, S. R. Vitiligo and pernicious anemia presenting as congestive heart failure. *Cutis* **46**(3), 268–270 (1990). 2225934

[20] Pelosio, A. et al. Pernicious anemia, vitiligo and positive antiglobulin test: An unusual association. *Haematologica* **74**(5), 499–501 (1989). 2511121

[21] Grimes, P. E. et al. Topical tacrolimus therapy for vitiligo: Therapeutic responses and skin messenger RNA expression of proinflammatory cytokines. *J. Am. Acad. Dermatol.* **51**(1), 52–61 (2004). 15243524 10.1016/j.jaad.2003.12.031

[22] Harris, J. E. et al. A mouse model of vitiligo with focused epidermal depigmentation requires IFN-gamma for autoreactive CD8(+) T-cell accumulation in the skin. *J. Invest. Dermatol.* **132**(7), 1869–1876 (2012). 22297636 10.1038/jid.2011.463 PMC3343174

[23] Yang, L. et al. Interferon-gamma inhibits melanogenesis and induces apoptosis in melanocytes: A pivotal role of CD8 + Cytotoxic T lymphocytes in vitiligo. *Acta Derm Venereol.* **95**(6), 664–670 (2015). 25721262 10.2340/00015555-2080

[24] Ng, C. Y. et al. Skin interstitial fluid and plasma multiplex cytokine analysis reveals IFN-gamma signatures and granzyme B as useful biomarker for activity, severity and prognosis assessment in vitiligo. *Front. Immunol.* **13**, 872458 (2022). 35464413 10.3389/fimmu.2022.872458 PMC9021541

[25] Shi, F. & Erf, G. F. IL-21, and IL-10 co-expression in evolving autoimmune vitiligo lesions of Smyth line chickens. *J. Invest. Dermatol.* **132**(3 Pt 1), 642–649 (2012). 22113479 10.1038/jid.2011.377 PMC3278581

[26] Wankowicz-Kalinska, A. et al. Immunopolarization of CD4 + and CD8 + T cells to type-1-like is associated with melanocyte loss in human vitiligo. *Lab. Invest.* **83**(5), 683–695 (2003). 12746478 10.1097/01.lab.0000069521.42488.1b

[27] Iwamoto, I. et al. Interferon gamma regulates antigen-induced eosinophil recruitment into the mouse airways by inhibiting the infiltration of CD4 + T cells. *J. Exp. Med.* **177**(2), 573–576 (1993). 8093895 10.1084/jem.177.2.573 PMC2190892

[28] Miyamasu, M. et al. Th1-derived cytokine IFN-gamma is a potent inhibitor of eotaxin synthesis in vitro. *Int. Immunol.* **11**(6), 1001–1004 (1999). 10360975 10.1093/intimm/11.6.1001

[29] Fukuda, K. et al. Inhibition of eotaxin expression in human corneal fibroblasts by interferon-gamma. *Int. Arch. Allergy Immunol.* **129**(2), 138–144 (2002). 12403931 10.1159/000065880

[30] Sato, T. et al. IFN-gamma-induced SOCS-1 regulates STAT6-dependent eotaxin production triggered by IL-4 and TNF-alpha. *Biochem. Biophys. Res. Commun.* **314**(2), 468–475 (2004). 14733929 10.1016/j.bbrc.2003.12.124

[31] Wang, X. X. et al. Increased expression of CXCR3 and its ligands in patients with vitiligo and CXCL10 as a potential clinical marker for vitiligo. *Br. J. Dermatol.* **174**(6), 1318–1326 (2016). 26801009 10.1111/bjd.14416

[32] Xanthou, G. et al. CCR3 functional responses are regulated by both CXCR3 and its ligands CXCL9, CXCL10 and CXCL11. *Eur. J. Immunol.* **33**(8), 2241–2250 (2003). 12884299 10.1002/eji.200323787

[33] Haley, K. J. et al. Overexpression of eotaxin and the CCR3 receptor in human atherosclerosis: Using genomic technology to identify a potential novel pathway of vascular inflammation. *Circulation* **102**(18), 2185–2189 (2000). 11056090 10.1161/01.cir.102.18.2185

[34] Economou, E. et al. Chemokines in patients with ischaemic heart disease and the effect of coronary angioplasty. *Int. J. Cardiol.* **80**(1), 55–60 (2001). 11532547 10.1016/s0167-5273(01)00454-5

[35] Kaehler, J. et al. Association between eotaxin (CCL11), C-reactive protein, and antimicrobial antibodies in patients undergoing coronary angioplasty. *J. Investig Med.* **54**(8), 446–454 (2006). 17169268 10.2310/6650.2006.06025

[36] Emanuele, E. et al. Association of plasma eotaxin levels with the presence and extent of angiographic coronary artery disease. *Atherosclerosis* **186**(1), 140–145 (2006). 16084515 10.1016/j.atherosclerosis.2005.07.002

[37] Kodali, R. B. et al. CCL11 (Eotaxin) induces CCR3-dependent smooth muscle cell migration. *Arterioscler. Thromb. Vasc Biol.* **24**(7), 1211–1216 (2004). 15130922 10.1161/01.ATV.0000131654.90788.f5

[38] Salcedo, R. et al. Eotaxin (CCL11) induces in vivo angiogenic responses by human CCR3 + endothelial cells. *J. Immunol.* **166**(12), 7571–7578 (2001). 11390513 10.4049/jimmunol.166.12.7571

[39] Schwartz, S. M. Smooth muscle migration in atherosclerosis and restenosis. *J. Clin. Invest.* **100**(11 Suppl), S87–S89 (1997). 9413408

[40] Gencer, S. et al. Inflammatory chemokines in atherosclerosis. *Cells* **10**(2) (2021). 10.3390/cells10020226 PMC7911854 33503867

[41] Zernecke, A. & Weber, C. Chemokines in the vascular inflammatory response of atherosclerosis. *Cardiovasc. Res.* **86**(2), 192–201 (2010). 20007309 10.1093/cvr/cvp391

[42] Buhl, T. & Schon, M. P. Peeking into immunoregulatory effects of phototherapy. *Exp. Dermatol.* **25**(7), 511–512 (2016). 27060231 10.1111/exd.13020

[43] Li, X. Y. et al. Weak UVB irradiation promotes macrophage M2 polarization and stabilizes atherosclerosis. *J. Cardiovasc. Transl Res.* **15**(4), 855–864 (2022). 34811697 10.1007/s12265-021-10189-7 PMC9622510

[44] Sasaki, N. et al. UVB exposure prevents atherosclerosis by regulating Immunoinflammatory responses. *Arterioscler. Thromb. Vasc Biol.* **37**(1), 66–74 (2017). 27765767 10.1161/ATVBAHA.116.308063

[45] Song, Z. et al. Clinically applicable histopathological diagnosis system for gastric cancer detection using deep learning. *Nat. Commun.* **11**(1), 4294 (2020). 32855423 10.1038/s41467-020-18147-8 PMC7453200

[46] Kabir, H. et al. Automated Estimation of cementitious sorptivity via computer vision. *Nat. Commun.* **15**(1), 9935 (2024). 39548066 10.1038/s41467-024-53993-w PMC11568162

[47] Jin, Y. et al. Genome-wide association studies of autoimmune vitiligo identify 23 new risk loci and highlight key pathways and regulatory variants. *Nat. Genet.* **48**(11), 1418–1424 (2016). 27723757 10.1038/ng.3680 PMC5120758

[48] Zhao, J. H. et al. Genetics of Circulating inflammatory proteins identifies drivers of immune-mediated disease risk and therapeutic targets. *Nat. Immunol.* **24**(9), 1540–1551 (2023). 37563310 10.1038/s41590-023-01588-w PMC10457199

[49] Hartwig, F. P., Davey, S. G. & Bowden, J. Robust inference in summary data Mendelian randomization via the zero modal pleiotropy assumption. *Int. J. Epidemiol.* **46**(6), 1985–1998 (2017). 29040600 10.1093/ije/dyx102 PMC5837715

