# Supplementary material for: Associations and mediators between vitiligo and cardiovascular diseases: a Mendelian randomization study
Source: Sci Rep. 2025 Apr 1;15:11110. doi: 10.1038/s41598-025-95638-y (PMC11961564; doi:10.1038/s41598-025-95638-y)
Supplement: Supplementary file 1 — Supplementary Material 1 [file 41598_2025_95638_MOESM1_ESM.docx]

Supplementary information

Associations and mediators between vitiligo and cardiovascular diseases: A Mendelian randomization study

Xiaoyan Zhang^1, #^, Lei Pu^1, #^, Cheng Pu^2^, Qian He^3,^ *

^1^The key Laboratory of Adolescent Health Assessment and Exercise Intervention of the Ministry of Education, East China Normal University, Shanghai 200241, P.R. China

^2^School of Martial Arts, Shanghai University of Sport, Shanghai 200438, P.R. China

^3^Preventive Medicine Department, Suzhou Wujiang District Second People's Hospital, Jiangsu 215221, P.R. China

*** Correspondence author**

Qian He.

Preventive Medicine Department, Suzhou Wujiang District Second People's Hospital, 999 DaChun Road, Jiangsu 215221, P.R. China


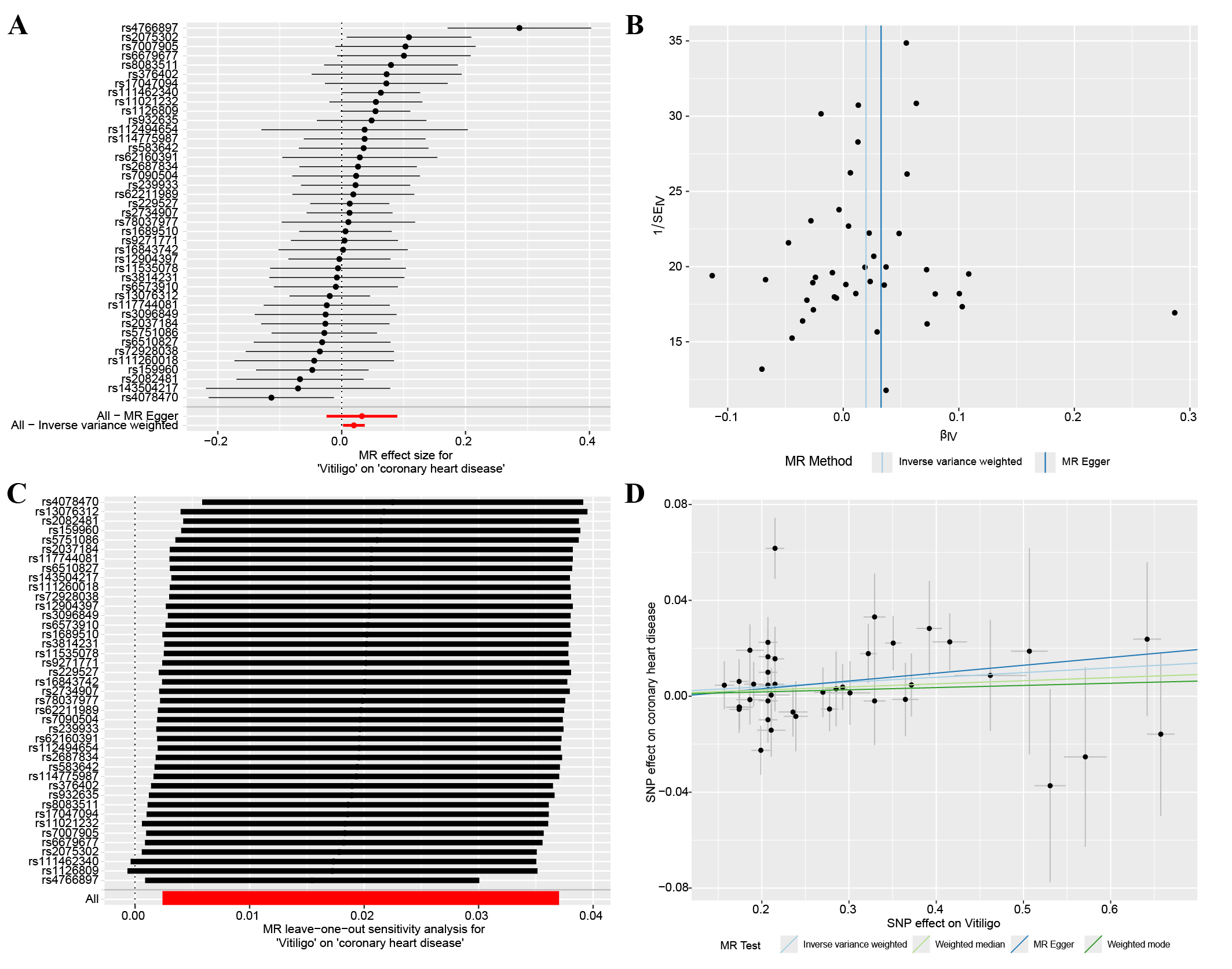


Figure S1. Association between vitiligo and coronary heart disease. (A) The forest plot showed that vitiligo increased the risk of coronary heart disease. (B) The funnel plot showed no significant bias. (C) The leave-one-out analysis showed that the results were robust. (D) The intercepts of different MR methods tended towards zero.


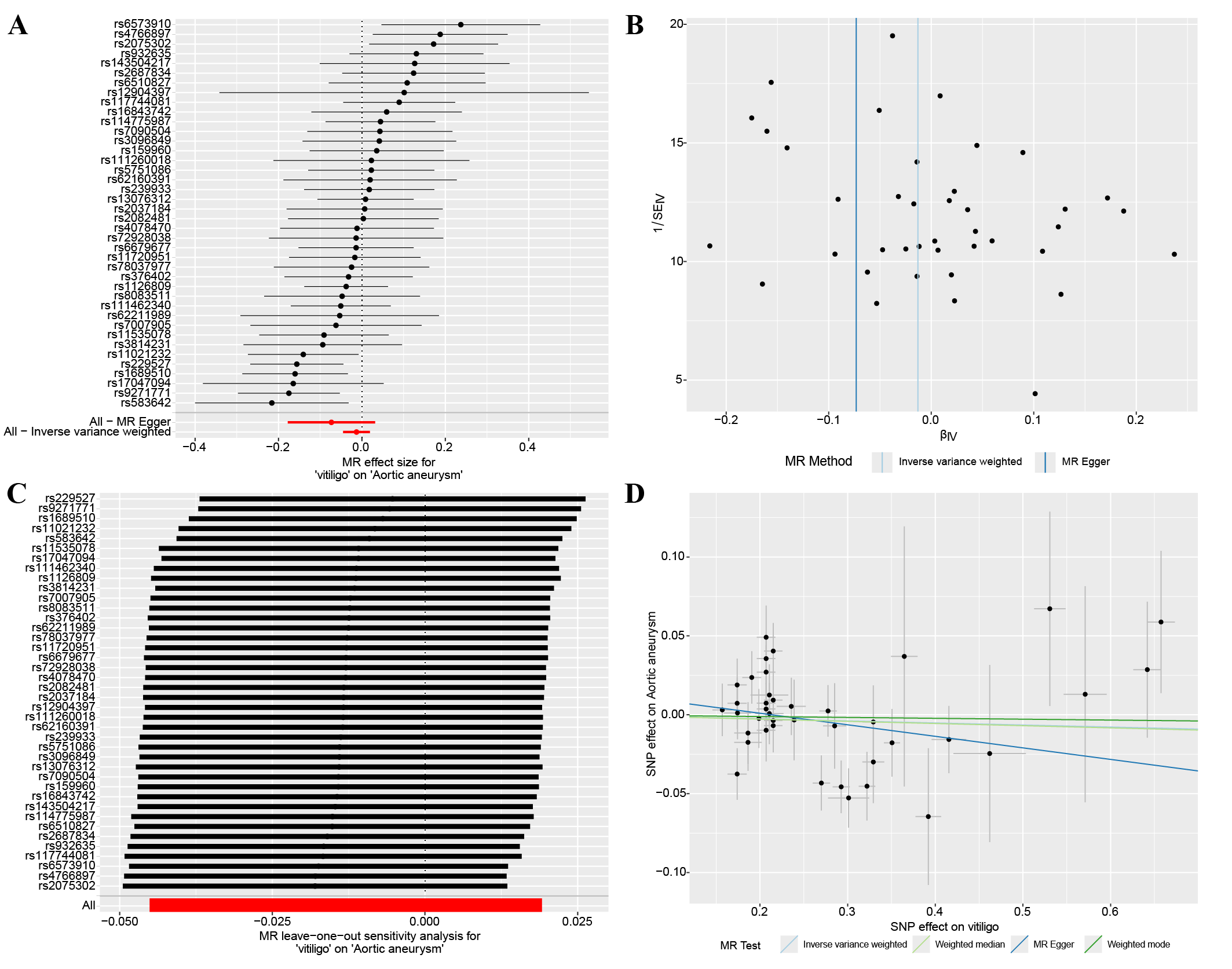


Figure S2. Association between vitiligo and aortic aneurysm. (A) The forest plot showed no significant association. (B) The funnel plot showed no significant bias. (C) The leave-one-out analysis showed that the results were robust. (D) The intercepts of different MR methods tended towards zero.


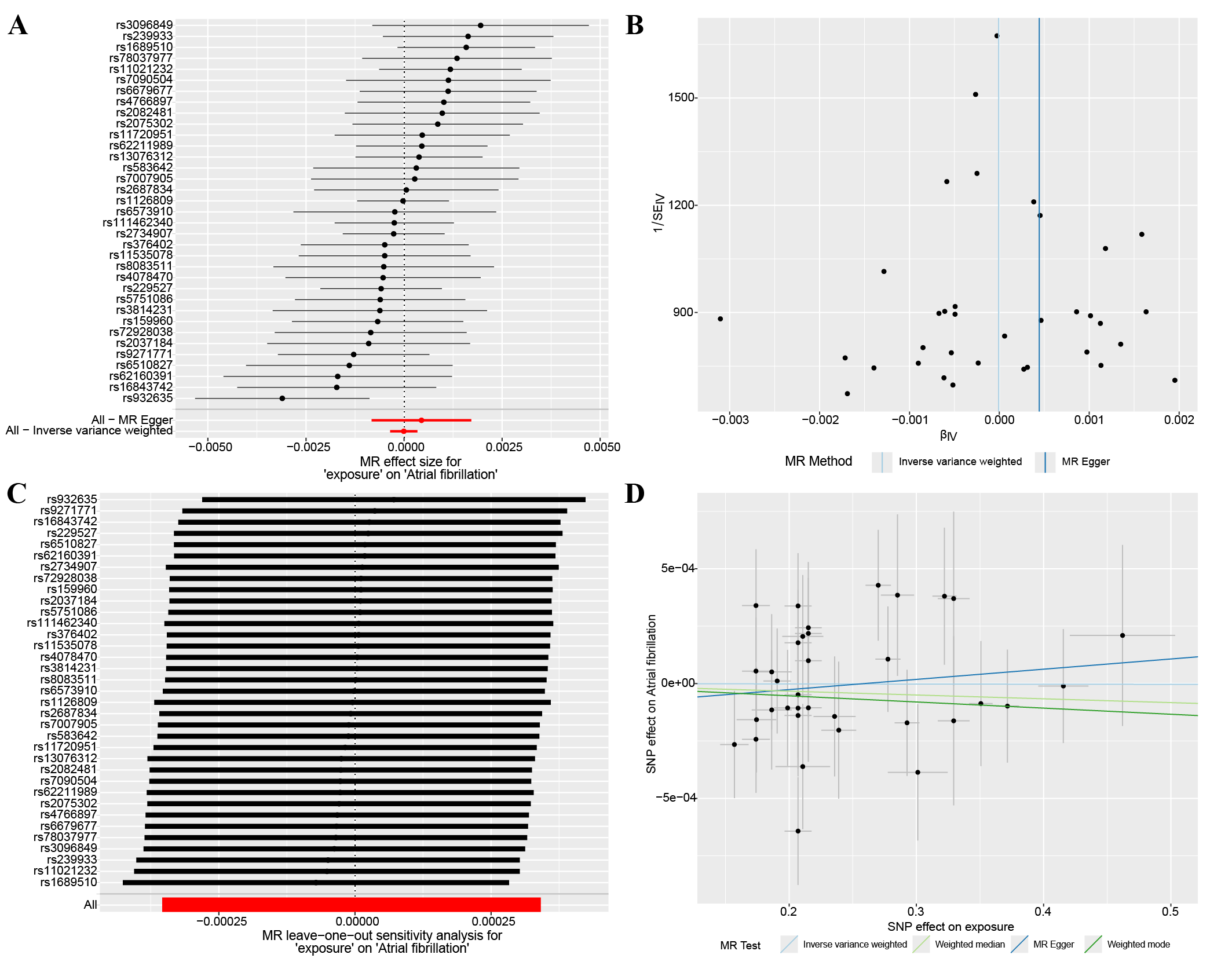


Figure S3. Association between vitiligo and atrial fibrillation. (A) The forest plot showed no significant association. (B) The funnel plot showed no significant bias. (C) The leave-one-out analysis showed that the results were robust. (D) The intercepts of different MR methods tended towards zero.


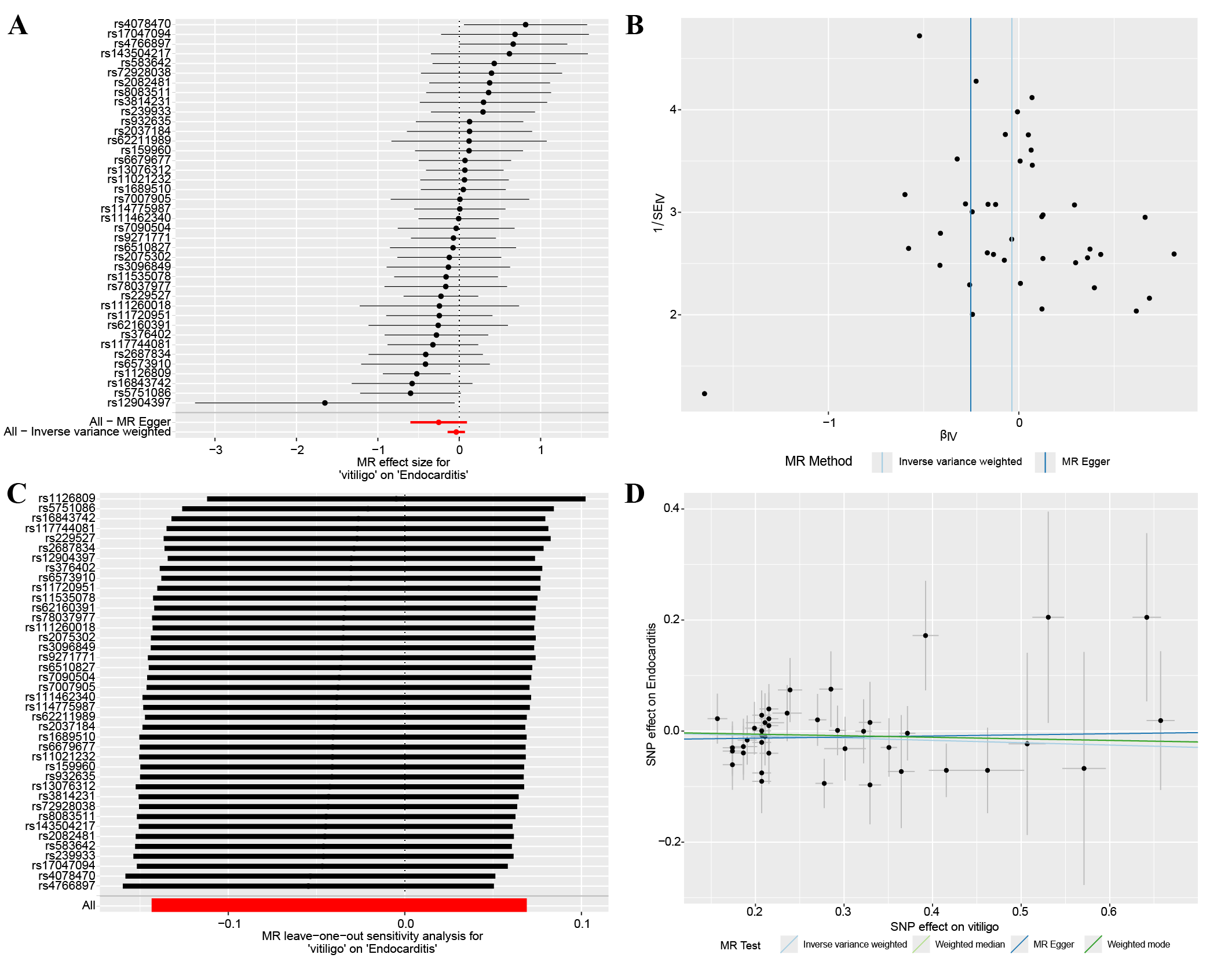


Figure S4. Association between vitiligo and endocarditis. (A) The forest plot showed no significant association. (B) The funnel plot showed no significant bias. (C) The leave-one-out analysis showed that the results were robust. (D) The intercepts of different MR methods tended towards zero.


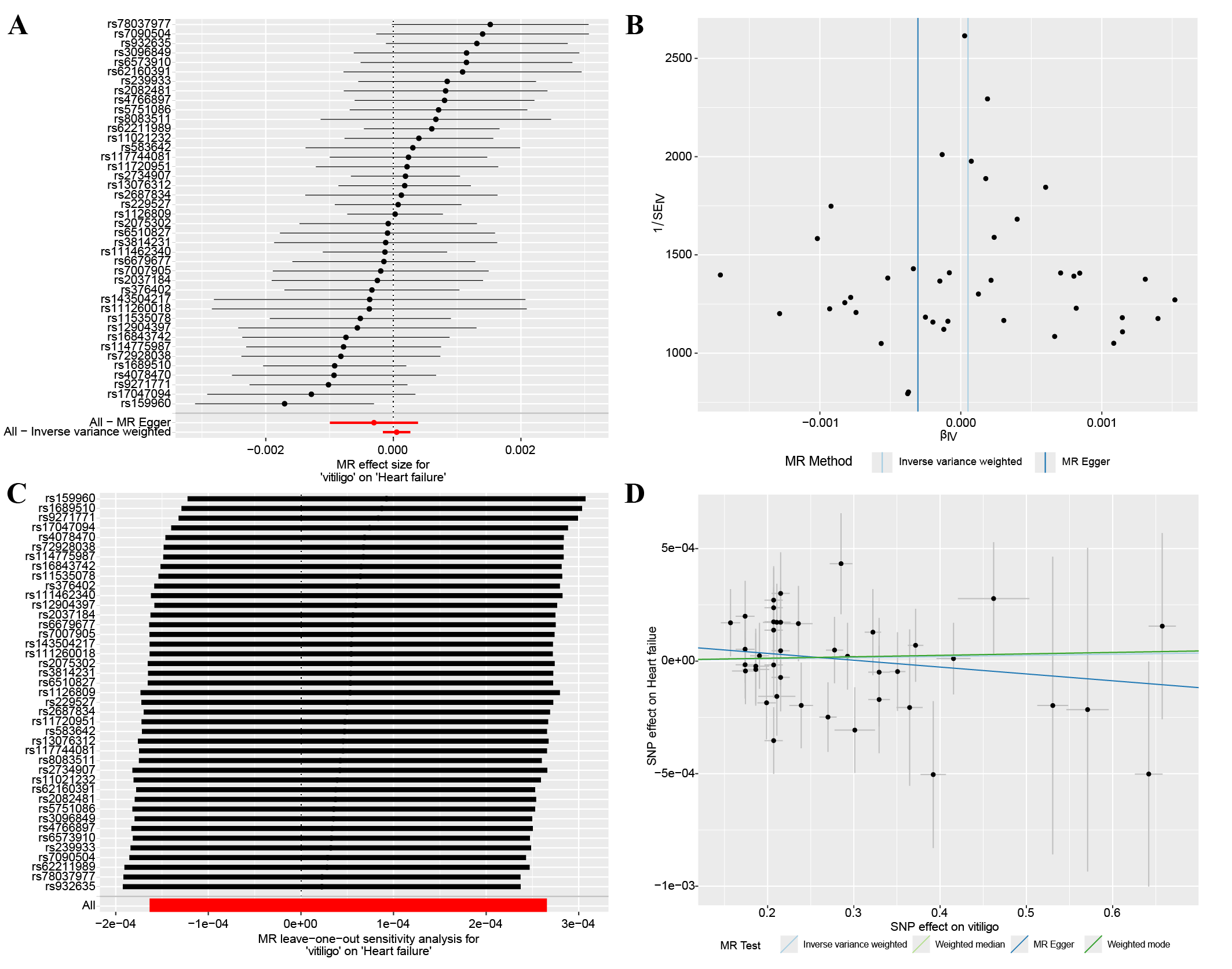


Figure S5. Association between vitiligo and heart failure. (A) The forest plot showed no significant association. (B) The funnel plot showed no significant bias. (C) The leave-one-out analysis showed that the results were robust. (D) The intercepts of different MR methods tended towards zero.


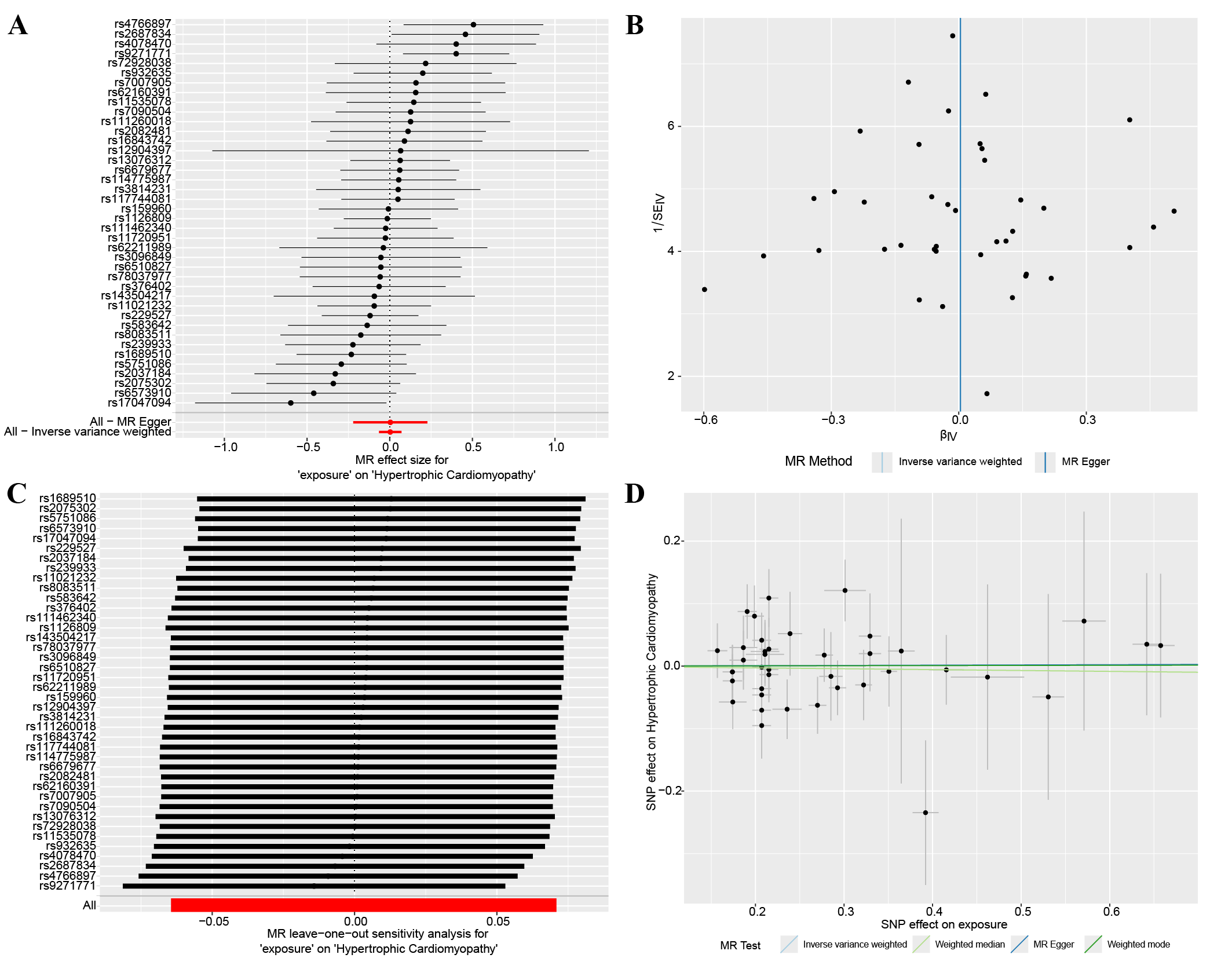


Figure S6. Association between vitiligo and hypertrophic cardiomyopathy. (A) The forest plot showed no significant association. (B) The funnel plot showed no significant bias. (C) The leave-one-out analysis showed that the results were robust. (D) The intercepts of different MR methods tended towards zero.


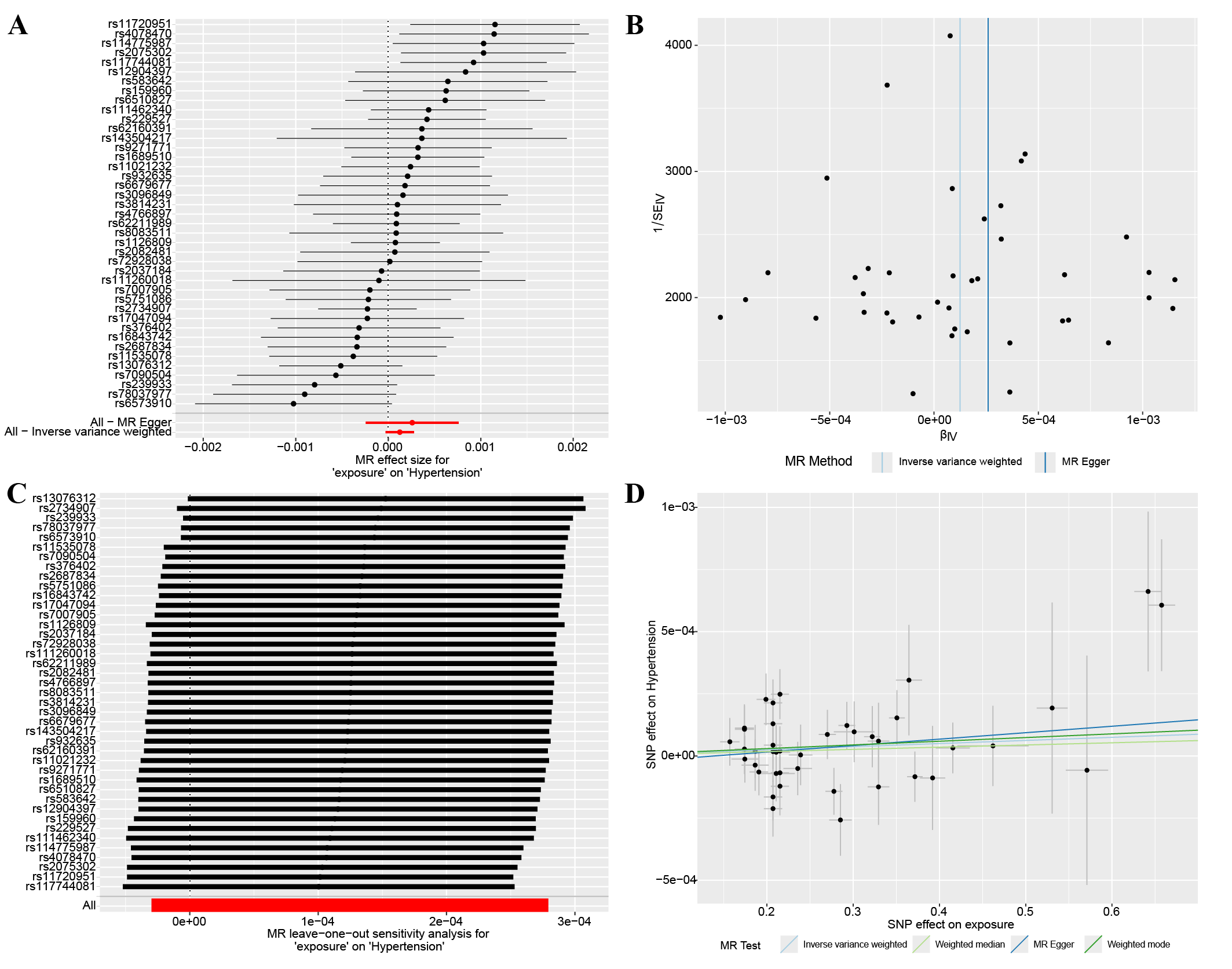


Figure S7. Association between vitiligo and hypertension. (A) The forest plot showed no significant association. (B) The funnel plot showed no significant bias. (C) The leave-one-out analysis showed that the results were robust. (D) The intercepts of different MR methods tended towards zero.


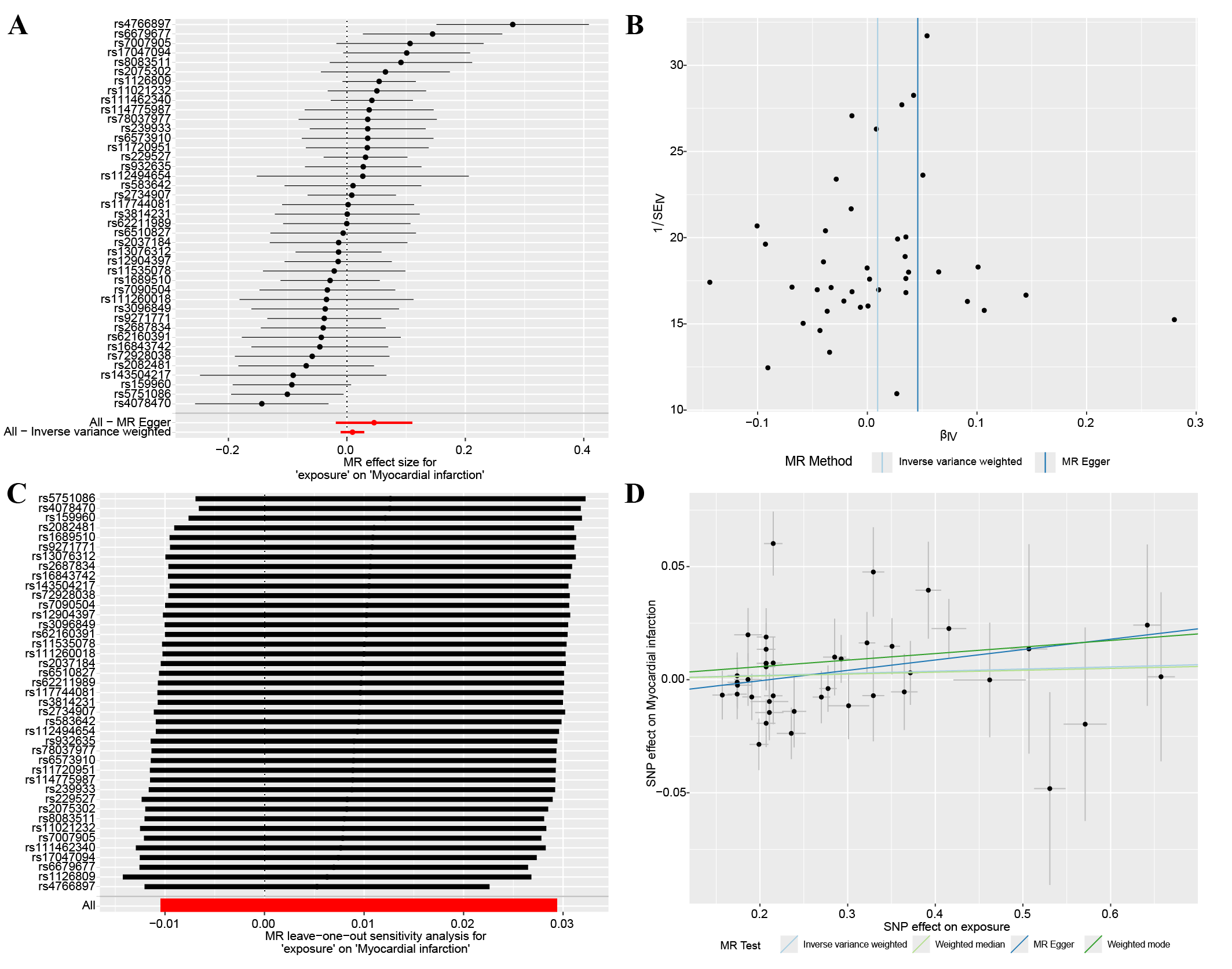


Figure S8. Association between vitiligo and myocardial infarction. (A) The forest plot showed no significant association. (B) The funnel plot showed no significant bias. (C) The leave-one-out analysis showed that the results were robust. (D) The intercepts of different MR methods tended towards zero.


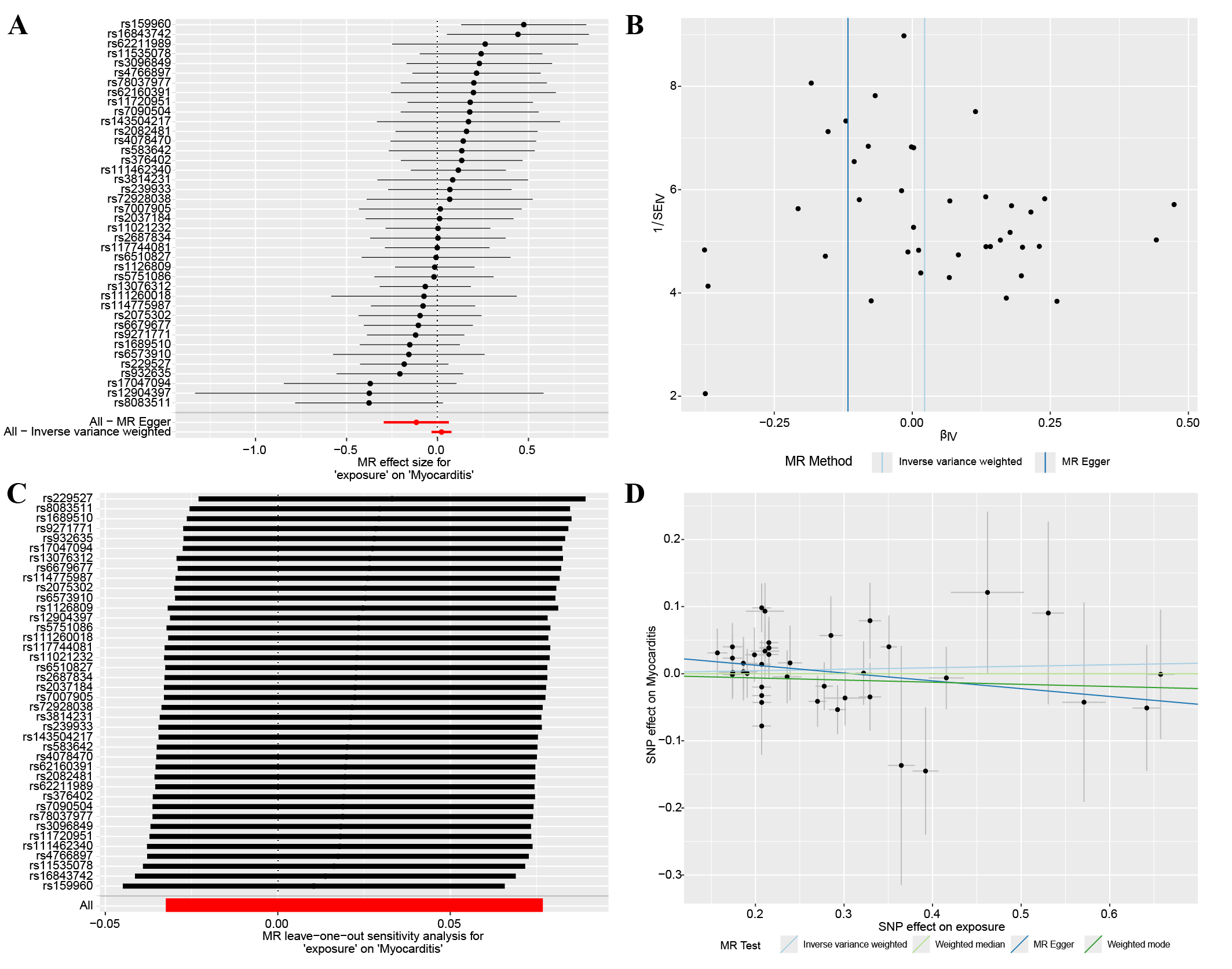


Figure S9. Association between vitiligo and myocarditis. (A) The forest plot showed no significant association. (B) The funnel plot showed no significant bias. (C) The leave-one-out analysis showed that the results were robust. (D) The intercepts of different MR methods tended towards zero.


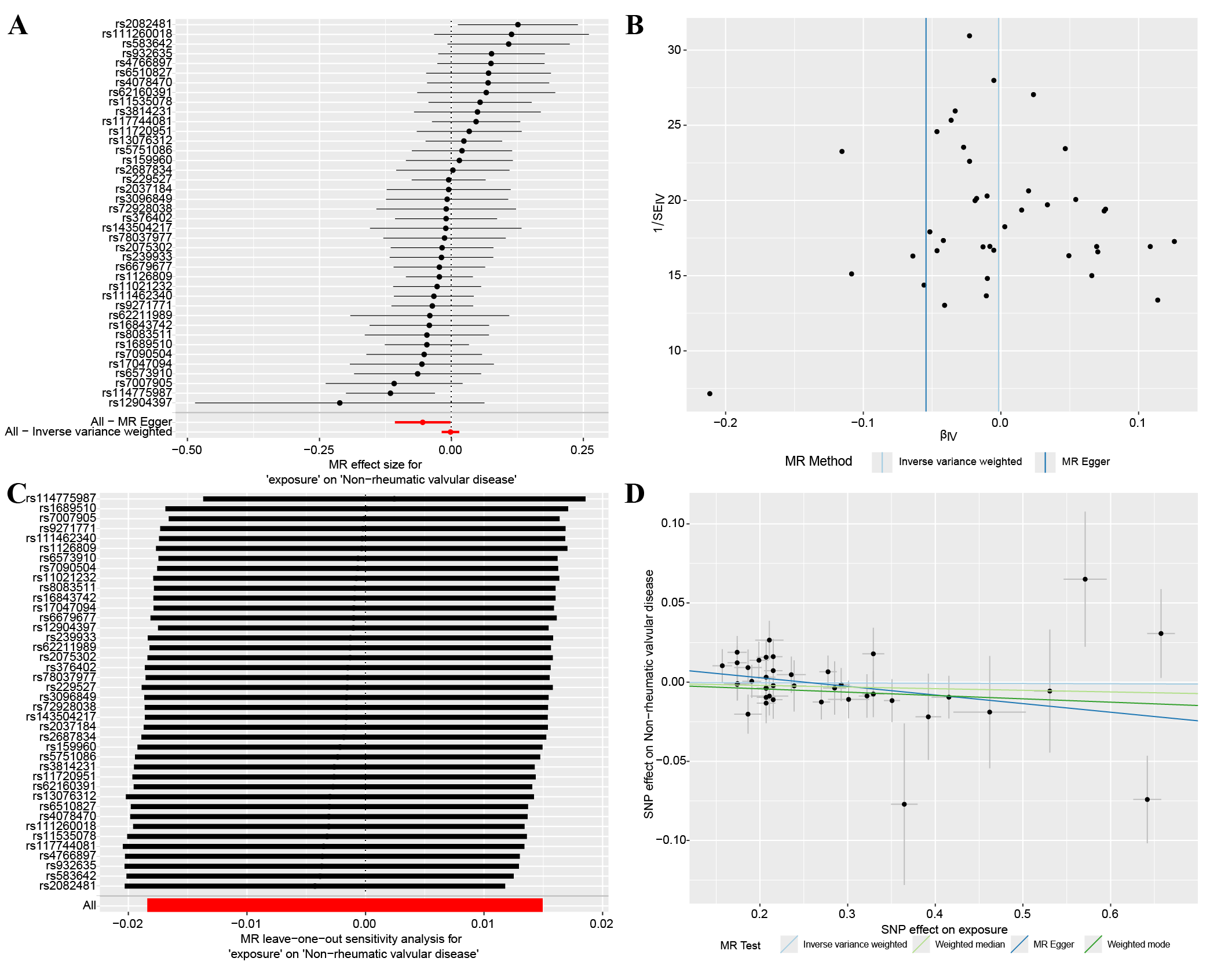


Figure S10. Association between vitiligo and non-rheumatic valvular disease. (A) The forest plot showed no significant association. (B) The funnel plot showed no significant bias. (C) The leave-one-out analysis showed that the results were robust. (D) The intercepts of different MR methods tended towards zero.


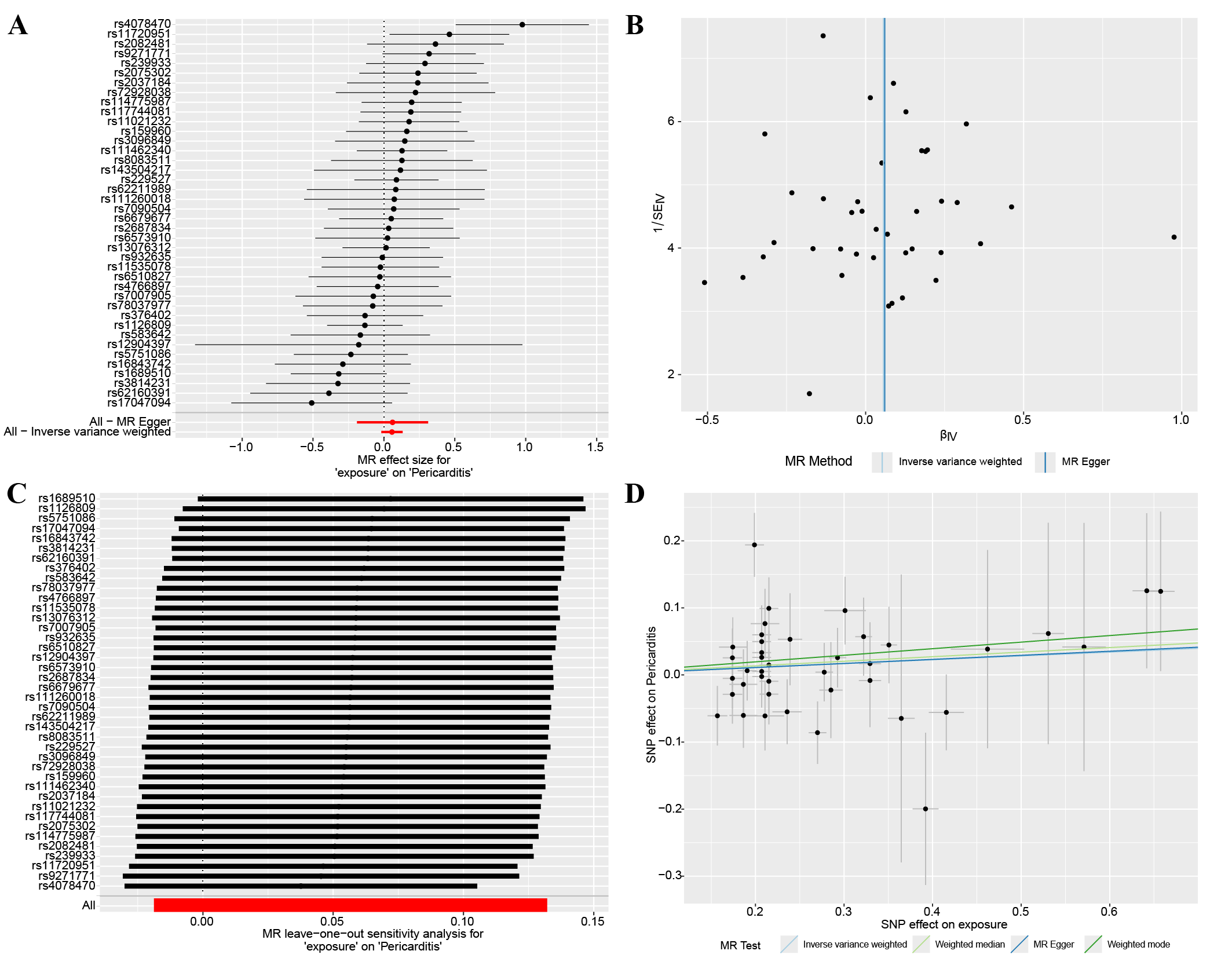


Figure S11. Association between vitiligo and pericarditis. (A) The forest plot showed no significant association. (B) The funnel plot showed no significant bias. (C) The leave-one-out analysis showed that the results were robust. (D) The intercepts of different MR methods tended towards zero.


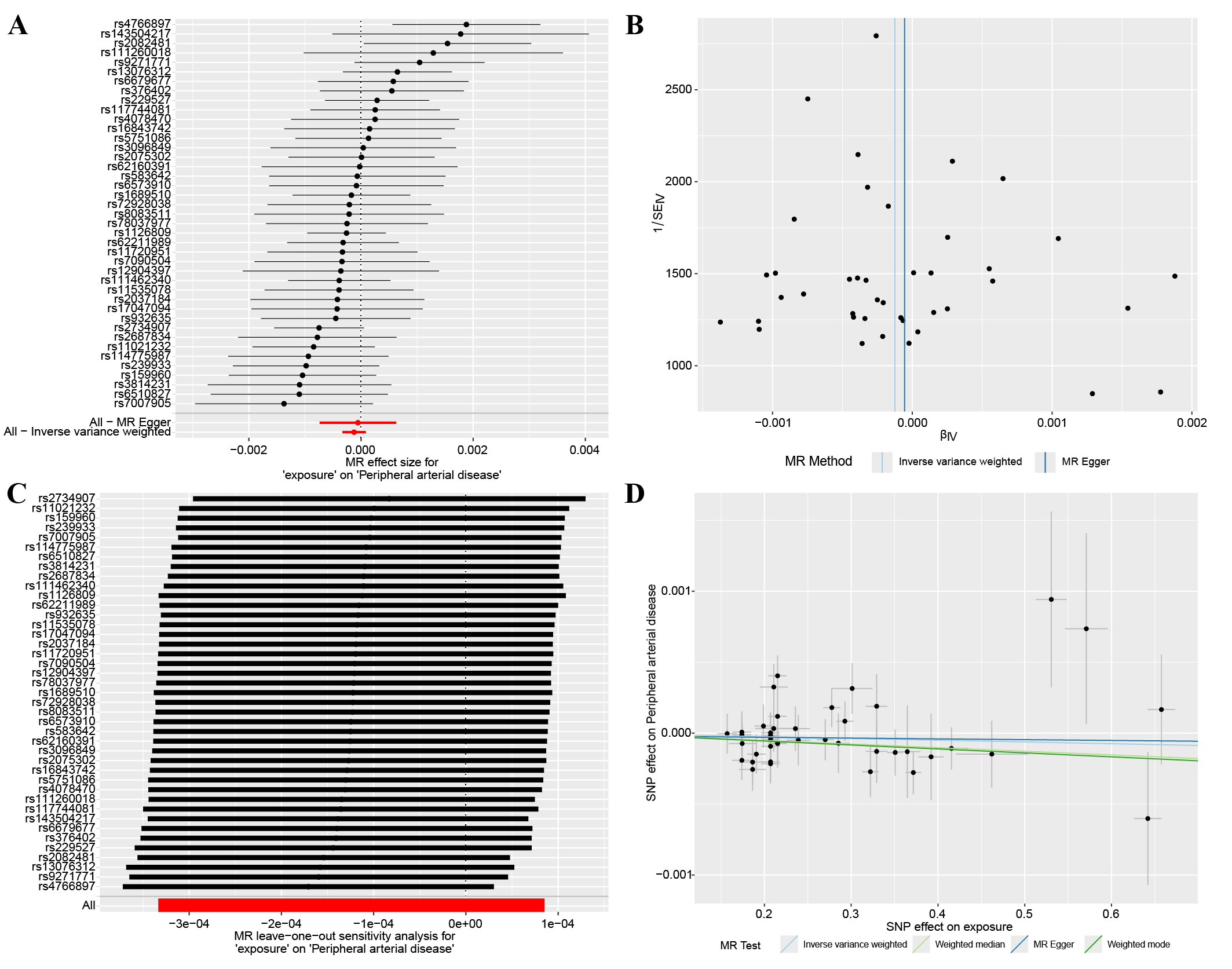


Figure S12. Association between vitiligo and peripheral arterial disease. (A) The forest plot showed no significant association. (B) The funnel plot showed no significant bias. (C) The leave-one-out analysis showed that the results were robust. (D) The intercepts of different MR methods tended towards zero.


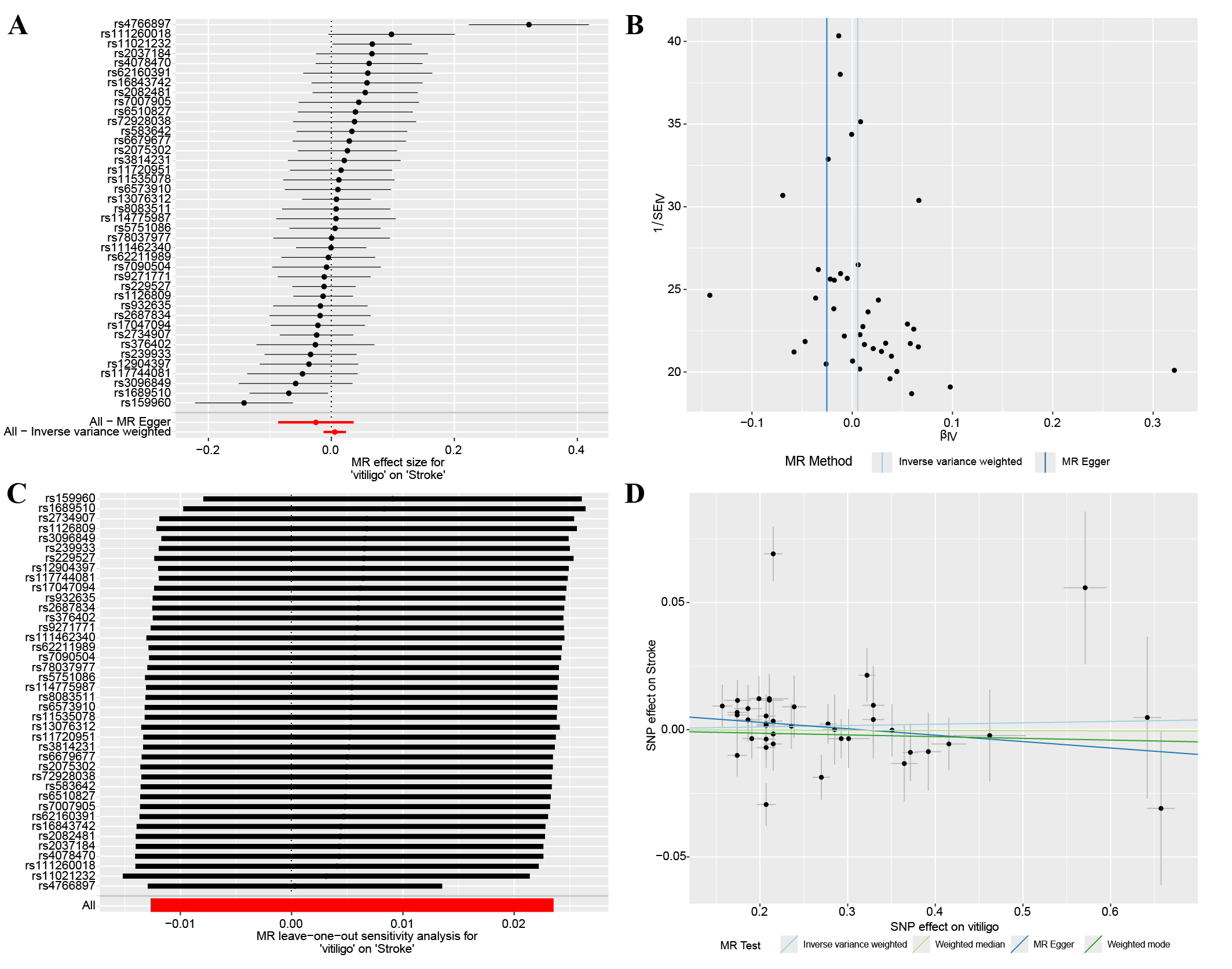


Figure S13. Association between vitiligo and stroke. (A) The forest plot showed no significant association. (B) The funnel plot showed no significant bias. (C) The leave-one-out analysis showed that the results were robust. (D) The intercepts of different MR methods tended towards zero.


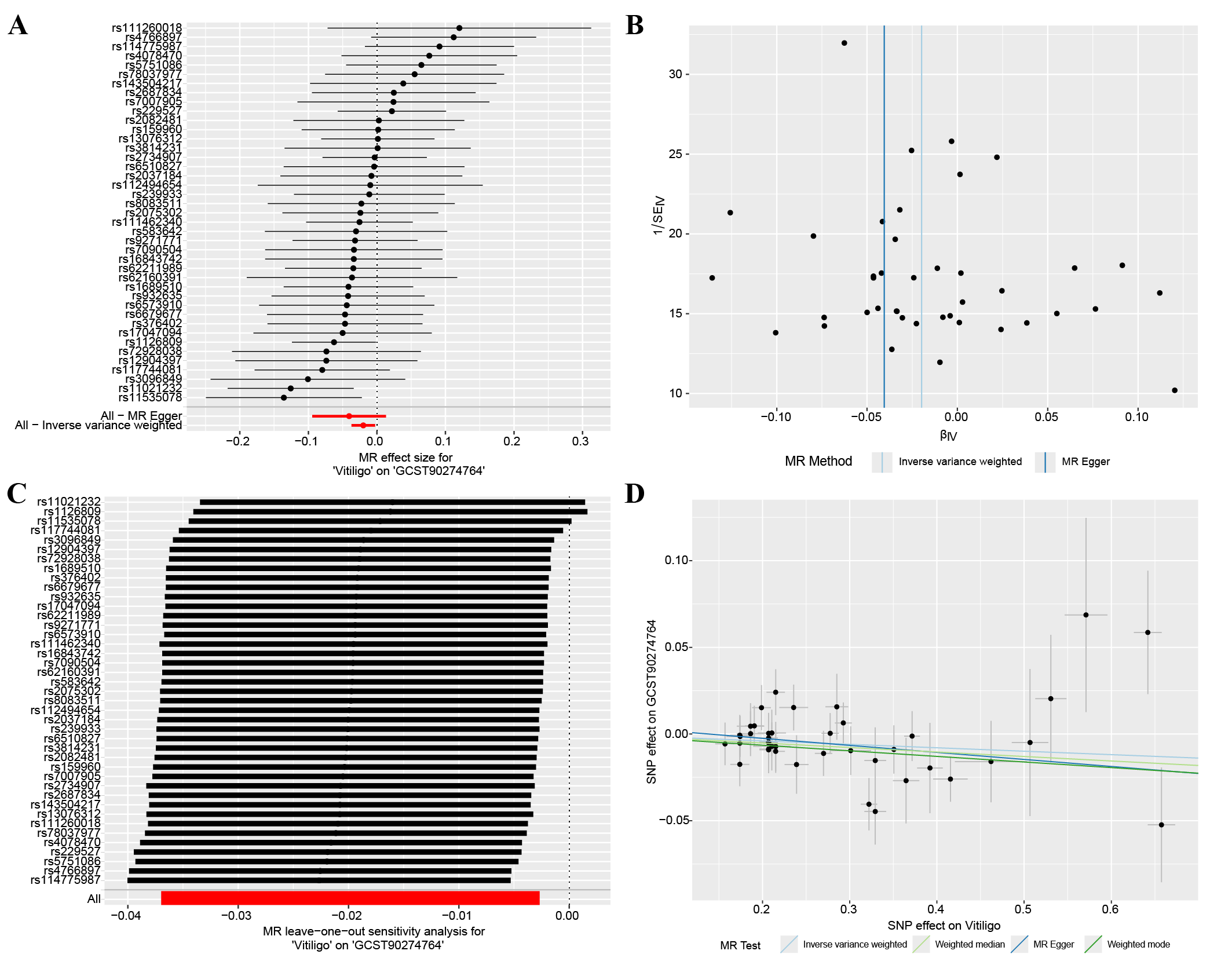


Figure S14. Association between vitiligo and CCL11. (A) The forest plot showed that vitiligo reduced CCL11 levels. (B) The funnel plot showed no significant bias. (C) The leave-one-out analysis showed that the results were robust. (D) The intercepts of different MR methods tended towards zero.


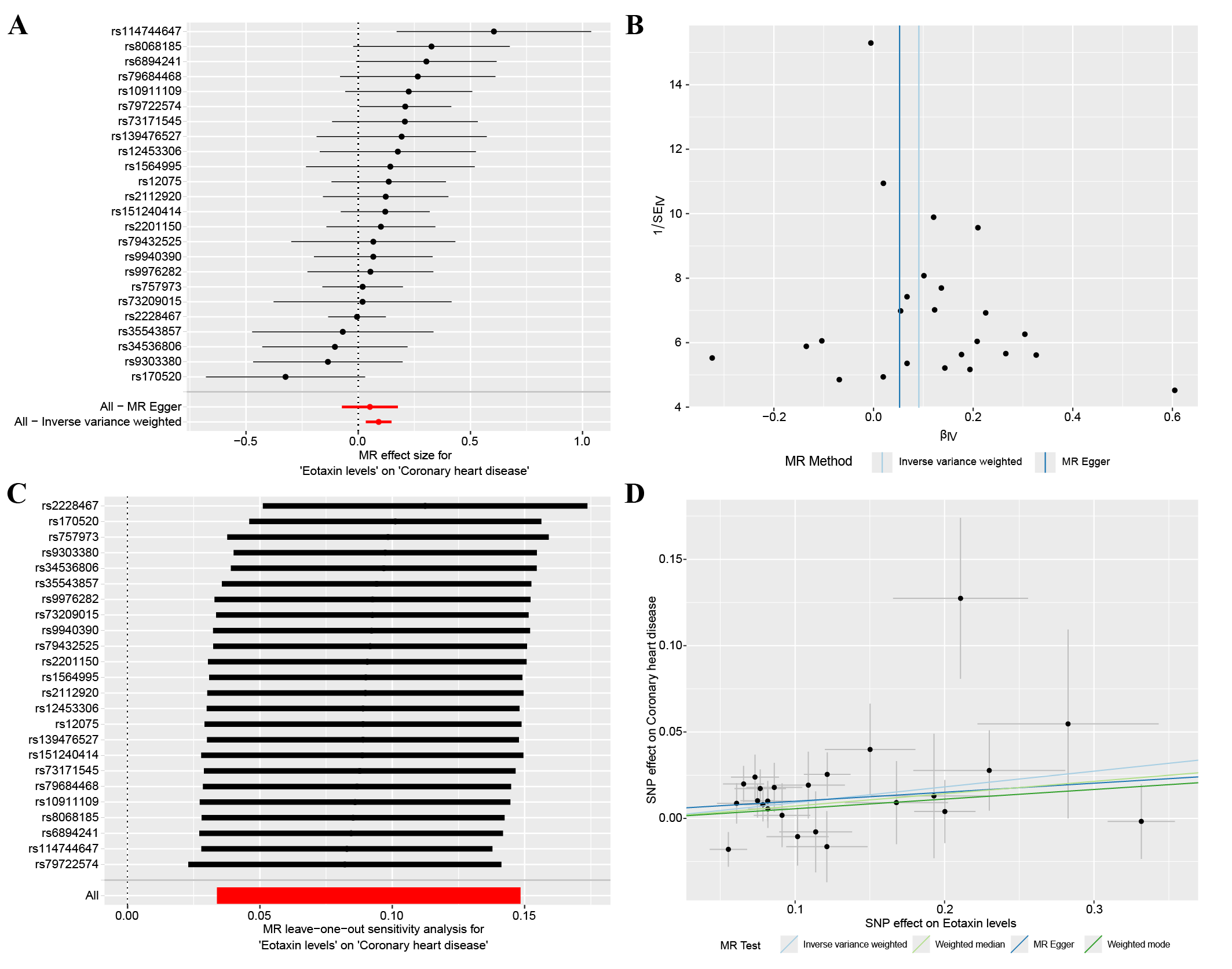


Figure S15. Association between CCL11 and coronary heart disease. (A) The forest plot showed that CCL11 increased CHD risk. (B) The funnel plot showed no significant bias. (C) The leave-one-out analysis showed that the results were robust. (D) The intercepts of different MR methods tended towards zero.
